# LaminA/C-dependent cellular senescence signaling promotes skeletal muscle atrophy and abnormalities in Parkinson’s disease

**DOI:** 10.1038/s41419-026-08962-0

**Published:** 2026-06-06

**Authors:** Jaydeep Sharma, Somnath Mondal, Krishna Singh Bisht, Ankit Biswas, Lakshmikanthan Panneerselvam, Tushar Kanti Maiti, Sam J. Mathew

**Affiliations:** 1 Developmental Genetics Laboratory, Regional Centre for Biotechnology (RCB), NCR Biotech Science Cluster, Faridabad, India; 2 Laboratory of Functional Proteomics, Regional Centre for Biotechnology (RCB), NCR Biotech Science Cluster, Faridabad, India

**Keywords:** Senescence, Parkinson's disease

## Abstract

Parkinson’s disease (PD) is a neurodegenerative disease affecting the central nervous system with effects on the skeletal muscle that entails detailed characterization. Several PD-associated motor symptoms, such as rigidity, movement delays and postural instability, involve the skeletal muscle. We used the human α-syn *A53T* mutant mouse model to characterize the PD-associated skeletal muscle abnormalities. These mice exhibit reduced muscle weight, myofiber size and grip strength at PD onset. Gain of slow muscle fibers at the expense of fast fibers, muscle stem cell number alterations, elevated fibrosis and neuromuscular junction degeneration were observed in these mice. Oxidative stress and DNA damage-associated pathways led to reduced levels of the nuclear membrane protein LaminA/C, causing accelerated cellular senescence in the A53T muscle. We identify a molecular pathway of senescence-associated secretory phenotype activating FoxO signaling, resulting in skeletal muscle loss in the A53T mice. Thus, increased oxidative stress and accumulated cellular senescence could underlie the PD-associated musculoskeletal defects, with potential therapeutic significance.

## Introduction

Parkinson’s disease (PD) is one of the most common neurodegenerative diseases, impairing both motor and non-motor functions. PD is caused by the progressive degeneration of the dopaminergic neurons in the substantia nigra region of the mid-brain. According to WHO reports, the prevalence of PD has doubled in the past 25 years, with over 8.5 million individuals affected globally [1, 2]. About 5–10% of PD is heritable, with the majority of PD cases classified as sporadic with no clearly attributable cause [3, 4]. Several proteins have been shown to contribute to PD pathology, affecting pathways regulating proteostasis, mitochondrial function, oxidative stress, etc. [4]. For instance, mutations in specific genes such as *Leucine-rich repeat kinase 2* (*LRRK2*), *α-synuclein* (*SNCA*) and several others are associated with PD [4]. Abnormal accumulation of proteinaceous aggregates called Lewy bodies, containing α-synuclein (α-syn) in neurons is another pathological feature of PD [4, 5]. α-syn mutations such as A30P, A53T and E46K have been reported in heritable PD; multiple mouse models of PD have been generated making use of these mutations.

While PD is primarily a disease of the central nervous system, many of the motor symptoms associated with PD, such as rigidity, slow movement (bradykinesia), postural instability and tremors involve the skeletal muscle. Although these symptoms have been described and are used extensively in the clinical diagnosis of PD, the underlying skeletal muscle abnormalities and molecular pathways have not been well studied. One of the key determinants of skeletal muscle function is its contractility, which in turn is regulated by several factors such as innervation, neuromuscular transmission, excitation-contraction coupling, muscle fiber type heterogeneity and myofilament function [6]. Skeletal muscle fiber type is regulated by hormonal and neural inputs, with fiber type alterations resulting from and contributing to various diseases [6]. Muscle fibers can broadly be slow (type I) or fast (type II); slow fibers are primarily oxidative in nature and express the myosin heavy chain (MyHC)-slow isoform, whereas the fast fibers are primarily glycolytic and express any of MyHC-IIa, -IIx or IIb isoforms. Since PD affects the nervous system and neural inputs through neuromuscular junctions (NMJs) that regulate muscle fiber type, it is likely that PD has effects on muscle fiber type. Previous studies indicate that NMJ dysfunction can lead to muscle fiber type alterations. For example, myasthenia gravis is an autoimmune disease characterized by antibodies targeting acetylcholine receptors at the NMJ, leading to structural and functional NMJ defects [7]. In a rat model for myasthenia gravis, a marked shift in fiber type with a significant increase in MyHC-slow+ type I fibers was observed [8]. Neural inputs are critical to muscle fiber type specification and maintenance, where the pattern, frequency and intensity of motor neuron firing play major roles. For instance, electrical stimulation of a fast muscle mimicking the firing patterns of slow motor neurons led to a transformation of the fast muscle to a slow phenotype, without changing the innervating motor neuron itself [9–11]. Limb-muscle biopsy from PD patients showed abnormal grouping of type I fibers with impaired mitochondrial function [12–14]. Some reports indicate that PD patients exhibit camptocormia- an axial postural deformity, and dropped head syndrome- resulting from severe weakness of neck muscles [15, 16]. Muscle biopsy of PD patients with camptocormia showed increased autophagy vacuoles, chronic inflammatory myopathy and abnormal mitochondrial function, including ragged-red fibers [15, 16].

The progressive loss of muscle mass with age is known as sarcopenia. A large proportion of PD patients exhibit sarcopenia and muscle atrophy [17], the molecular basis of which needs to be understood. One contributing factor could be cellular senescence, which is an irreversible arrest of the cell cycle due to diverse stress conditions. Cellular senescence has been reported to occur even in mitotically inactive cell types such as mature myofibers of the skeletal muscle and neurons in the nervous system. In aged mice, a subset of myofibers exhibits elevated p21 expression, a cellular senescence marker and exhibits a transcriptional profile characteristic of senescence [18]. Constitutive overexpression of p21 induced cellular senescence and associated pathology in the skeletal muscle [19]. Similar mechanisms mediated by the p21 pathway have been shown to operate in neurons, which are also post-mitotic [20, 21]. Elevated cellular senescence has been reported in the microglia and astrocytes of the central nervous system in PD, emerging as one of the major risk factors of PD [22–24]. Senescence markers such as p21 and p53 have been found to be upregulated in aged mice, indicating their possible role in age-associated muscle mass loss [25–27]. Senescent cells secrete pro-inflammatory signals, broadly known as senescence-associated secretory phenotype (SASP) [28, 29]. Skeletal muscle mass is tightly regulated by a balance between protein synthesis and degradation. Recent evidence suggests that SASPs contribute significantly to muscle mass loss [18]. Cellular stresses, such as oxidative stress characterized by the generation of reactive oxygen species, are accompanied by increased SASPs [30]. LaminA/C, a nuclear lamina protein essential for maintaining nuclear structure, DNA organization and DNA damage repair, is closely associated with cellular senescence and muscle function. Mutations in LaminA/C lead to premature aging syndromes such as progeria and muscle diseases such as Emery-Dreifuss muscular dystrophy [31]. Under different stress conditions in the muscle, the muscle-specific E3 ubiquitin ligases atrogin and MuRF1 get activated, leading to increased ubiquitination of muscle-specific proteins. Atrogin and MuRF1 are both regulated by the Akt-FoxO signaling axis, contributing to muscle atrophy [32, 33].

In this study, we have used the human α-syn *A53T* mutant mouse model to characterize the skeletal muscle defects associated with PD. These mice exhibit reduced body weight, muscle weight, myofiber size and grip strength at 16 months of age. Interestingly, increased activity levels and oxygen consumption were also seen in the A53T mice. Muscle fiber type alterations, with an increase in slow fibers and a decrease in fast MyHC-IIb+ and IIx+ fibers, muscle-dependent changes in satellite cell numbers and elevated fibrosis were observed in the A53T mice. The NMJs of A53T mice exhibited accumulation of α-syn and the pathogenic phospho-α-syn, leading to NMJ fragmentation and degeneration. Mass spectrometric analysis of diverse skeletal muscle types indicated that oxidative stress, DNA damage and related pathways were most affected in the A53T muscle. Elevated oxidative stress and DNA damage are known to lead to a reduction in expression of the nuclear membrane protein LaminA/C, which was found to be significantly downregulated in the skeletal muscle of A53T mice. Since reduced LaminA/C levels can result in cellular senescence, markers of cellular senescence, such as p21 and p53, were ascertained, which were upregulated in the A53T muscle. Accordingly, levels of SASPs such as tumor necrosis factor-α (TNF-α) and interleukin-6 (IL-6) were upregulated, leading to reduced insulin-like growth factor-1 (IGF-1) levels, activation of Forkhead Box O (FoxO) signaling, resulting in activation of the E3 ubiquitin ligases MuRF1 and atrogin and muscle loss. Thus, increased oxidative stress, DNA damage and accumulated cellular senescence could underlie the musculoskeletal defects in PD, which could be valuable therapeutic targets.

## Results

### A mouse model for Parkinson’s disease exhibits muscle defects

**Fig. 1:**
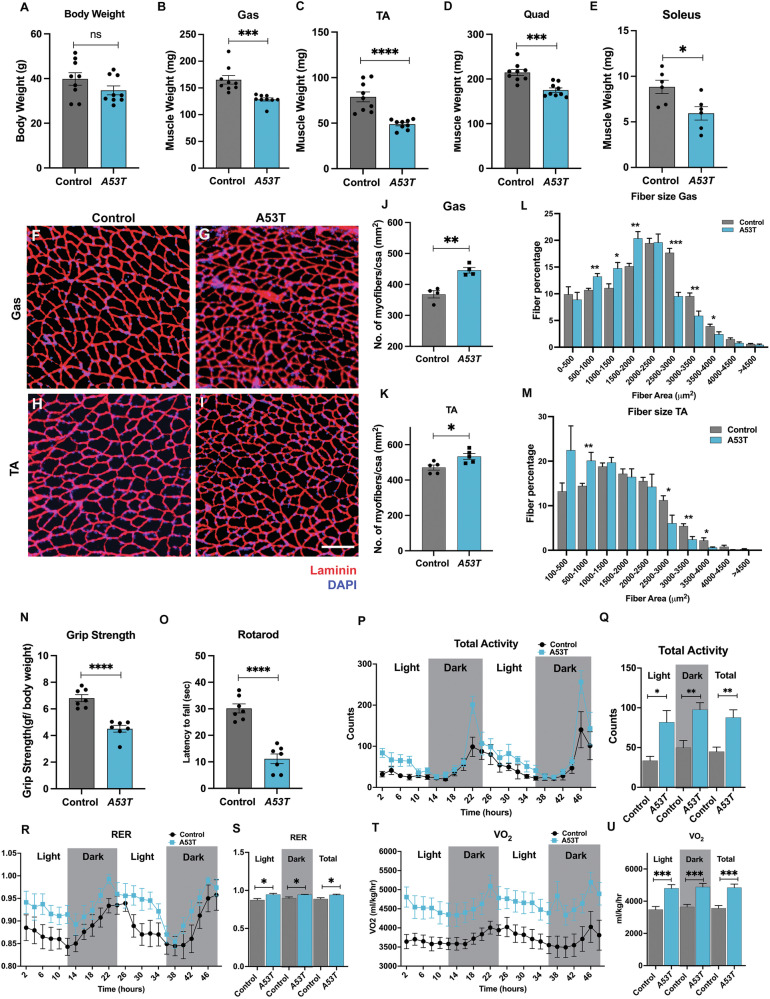
A53T mice exhibit muscle structural and functional defects. **A** Total body weight (grams) of non-transgenic (Control) and human α-Syn hemizygous (*A53T*^+/^^−^) female mice measured at 16 months of age. **B**–**E** Individual muscle weight (milligrams) of gastrocnemius (Gas), tibialis anterior (TA), quadriceps (Quad), and soleus of non-transgenic (Control) and human α-Syn hemizygous (*A53T*^+/^^−^) female mice measured at 16 months of age. **F**–**I** Representative fluorescent micrographs of transverse sections through the gastrocnemius (Gas) and TA muscle of non-transgenic (Control) and human α-Syn hemizygous (*A53T*^+/^^−^) mice measured at 16 months of age stained with Laminin (red), and DAPI (blue). **J**, **K** Quantification of the number of myofibers normalized to cross-sectional area (CSA) (mm^2^) through the gastrocnemius (Gas) and TA muscle of non-transgenic (Control) and human α-Syn hemizygous (*A53T*^+/^^−^) mice measured at 16 months of age. **L**, **M** Quantification of the number of myofibers grouped according to myofiber area through the gastrocnemius (Gas) and TA muscle of non-transgenic (Control) and human α-Syn hemizygous (*A53T*^+/^^−^) mice measured at 16 months of age (*n* = 4 mice per genotype). **N** Quantification of grip strength of non-transgenic (Control) and human α-Syn hemizygous (*A53T*^+/−^) female mice normalized to body weight (gf/gram body weight) at 16 months of age. **O** Rotarod analysis of 16-month-old non-transgenic (Control) and human α-Syn hemizygous (*A53T*^+/^^−^) female mice depicting the mean time of latency to fall (seconds) of individual mice. **P**, **Q** Graphs quantifying the hourly (**P**) and average (**Q**) total locomotor activity (X, Y and Z) of 16-month-old non-transgenic (Control) and human α-Syn hemizygous (*A53T*^+/^^−^) female mice (*n* = 6 mice per genotype). **R**, **S** Graphs quantifying the hourly (**R**) and average (**S**) respiratory exchange ratio (RER) of 16-month-old non-transgenic (Control) and human α-Syn hemizygous (*A53T*^+/^^−^) female mice (*n* = 6 mice per genotype). **T**, **U** Graphs quantifying the hourly (**T**) and average (**U**) volume of oxygen consumed (VO_2_) (ml/kg/hr) of 16-month-old non-transgenic (Control) and human α-Syn hemizygous (*A53T*^+/^^−^) female mice (*n* = 6 mice per genotype). Data information: Data are presented as mean ± SEM. Student’s *t* test was performed, with *p* ≤ 0.05 considered significant. Scale bar: 100 µm (**I**).

To study the abnormalities in a mouse model for PD, we used the human α-Syn hemizygous (*A53T*^+/−^) and littermate non-transgenic (control) mice, which show PD symptoms by 16 months of age [34]. The human α-Syn hemizygous *A53T*^+/^^−^ mice (referred to as A53T mice henceforth) did not exhibit any significant change in total body weight compared to controls in both females (Fig. 1A) and males (Fig. S1A) at 16 months. However, the A53T mice exhibited a clear >15% reduction in muscle weight, specifically in the gastrocnemius, tibialis anterior (TA), quadriceps and soleus muscles in females at 16 months (Fig. 1B-E). Similar results were also seen in male A53T mice at 16 months (Fig. S1B-E). Some of these muscles were taken as representative muscles of differing fiber type composition and metabolic characteristics. These results indicate that PD leads to a reduction in muscle weight in limb muscles of varying fiber type composition in mice. To characterize this, myofibers were grouped according to cross-sectional area (CSA), and the proportion of myofibers falling in each group was quantified. An increase in the proportion of smaller-sized fibers and a decrease in the proportion of larger-sized fibers was consistently observed across the gastrocnemius, TA, soleus and extensor digitorum longus (EDL) muscles in the A53T mice (Figs. 1F–I, L, M and S1F–I, L, M). The number of myofibers per unit area was increased in the gastrocnemius, TA, soleus and EDL muscles (Fig. 1J, K and S1J, K). These results confirm that the increase in the number of myofibers and decrease in the muscle weight are due to the reduction in myofiber size in the A53T mice. The A53T mice exhibited significantly reduced muscle function measured by grip strength normalized to body weight, compared to controls in females (Fig. 1N) and males (Fig. S1N). In rotarod assays, the A53T mice showed significantly reduced latency to fall compared to controls in females (Fig. 1O) and males (Fig. S1O). Next, we measured the activity and physiological parameters of the control and A53T mice using the Comprehensive Lab Animal Monitoring System (CLAMS). The A53T mice exhibited increased locomotor activity both during the light and dark cycles, resulting in increased total activity (Fig. 1P, Q). Increased activity was shown by the A53T mice in the X, Y and Z planes of the cage (Fig. S1P–U). Interestingly, the A53T mice also exhibited elevated respiratory exchange rate (RER) and increased oxygen consumption compared to controls (Fig. 1R–U). Expression of α-Syn and the pathogenic Phospho-α-Syn was confirmed in the gastrocnemius muscle of A53T mice by western blots (Fig. S1V, W). Interestingly, ex vivo muscle contractility assays using the TA muscle of A53T mice following electrical stimulation indicated markedly reduced absolute contractile force, specific force and maximum contractile force compared to controls (Fig. S1X–Y’). A significant increase in centralized myonuclei, indicating increased muscle damage, was observed in the A53T TA and gastrocnemius muscles, with no change in nuclear density (Fig. S1Z–AA). These results indicate that the A53T mouse model for PD exhibits muscle defects at the structural and functional level.

### Muscle fiber type proportions are altered in the A53T mice

**Fig. 2:**
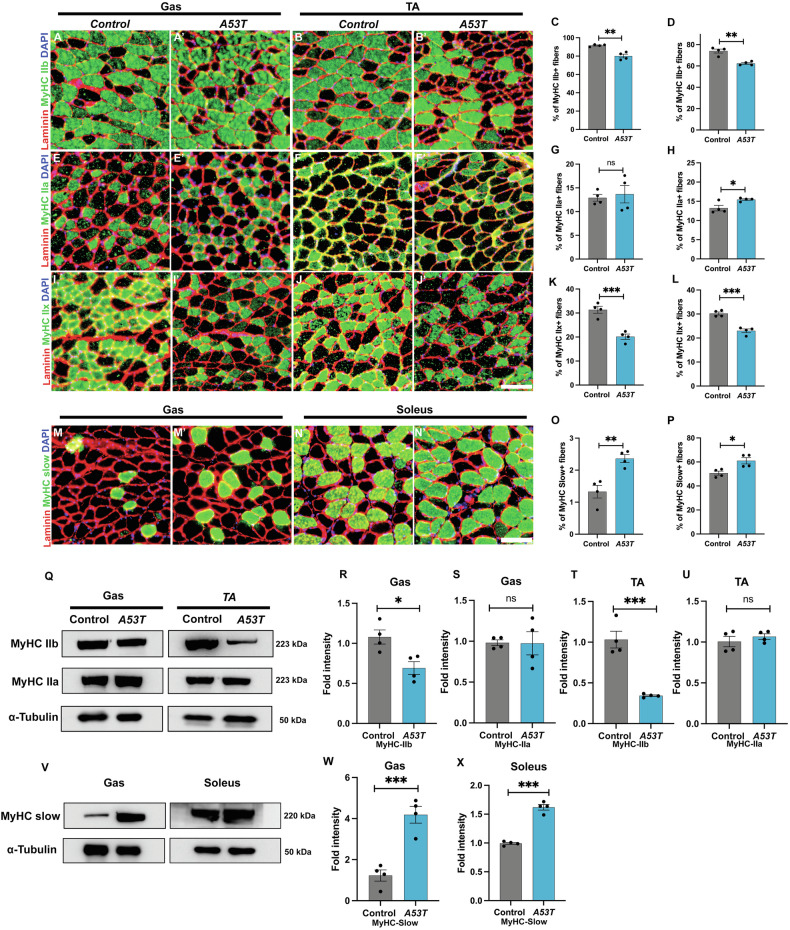
A53T mice exhibit alterations in muscle fiber type. **A**–**D** Representative fluorescent micrographs of transverse sections through the gastrocnemius (Gas) and tibialis anterior (TA) muscle of 16 months old non-transgenic (Control) and human α-Syn hemizygous (*A53T*^+/^^−^) mice labeled for Laminin (red), MyHC-IIb (green) and DAPI (blue) (**A**–**B**’), and quantification of the percentage of MyHC-IIb+ fibers (**C**, **D**). **E**–**H** Representative fluorescent micrographs of transverse sections through the gastrocnemius (Gas) and TA muscle of 16-month-old non-transgenic (Control) and human α-Syn hemizygous (*A53T*^+/^^−^) mice labeled for Laminin (red), MyHC-IIa (green) and DAPI (blue) (**E**–**F**’), and quantification of the percentage of MyHC-IIa+ fibers (**G**, **H**). **I**–**L** Representative fluorescent micrographs of transverse sections through the gastrocnemius (Gas) and TA muscle of 16-month-old non-transgenic (Control) and human α-Syn hemizygous (*A53T*^+/^^−^) mice labeled for Laminin (red), MyHC-IIx (green) and DAPI (blue) (**I**–**J**’), and quantification of the percentage of MyHC-IIx+ fibers (**K**, **L**). **M**–**P** Representative fluorescent micrographs of transverse sections through the gastrocnemius (Gas) and soleus muscle of 16-month-old non-transgenic (Control) and human α-Syn hemizygous (*A53T*^+/^^−^) mice labeled for Laminin (red), MyHC-slow (green) and DAPI (blue) (**M**–**N**’), and quantification of the percentage of MyHC-slow+ fibers (**O**, **P**). **Q**–**U** Representative western blots for MyHC-IIb, -IIa and α-tubulin (loading control) using protein lysates from the gastrocnemius (Gas) and TA muscle of 16-month-old non-transgenic (Control) and human α-Syn hemizygous (*A53T*^+/^^−^) mice (**Q**) and their densitometric quantification (**R**–**U**). **V**–**X** Representative western blots for MyHC-slow and α-tubulin (loading control) using protein lysates from the gastrocnemius (Gas) and soleus muscle of 16-month-old non-transgenic (Control) and human α-Syn hemizygous (*A53T*^+/^^−^) mice (**V**) and their densitometric quantification (**W**, **X**). Data information: Quantification of MyHC+ fibers is represented as a percentage of MyHC+ fibers normalized to total number of fibers. Data are presented as mean ± SEM. Student’s *t* test was performed, with *p* ≤ 0.05 considered significant. Scale bar: 100 µm (**J**’, **N**’).

Next, we wished to characterize the effect of PD on adult muscle fiber type. We carried out immunofluorescence labeling for adult MyHC isoforms on cross-sections through the gastrocnemius, TA, soleus and EDL muscles of control and A53T mice, at 16 months of age. The percentage of MyHC-IIb+ and MyHC-IIx+ fiber numbers compared to total fiber numbers was significantly reduced in A53T mice in the gastrocnemius and TA (Fig. 2A–D, I–L). Similar results were seen in the EDL muscle also (Fig. S2A–C, G-I). MyHC-IIa+ fiber numbers exhibited a marginal but significant increase in the TA muscle; however, there was no significant change in MyHC-IIa+ fiber proportion in the gastrocnemius or the EDL (Figs. 2E–H and S2D–F). Since the TA has relatively few MyHC-slow+ fibers, we characterized the effect of PD on MyHC-slow fibers in the gastrocnemius and soleus muscles, where we observed a significant increase in the percentage of MyHC-slow+ fibers in the A53T mice (Fig. 2M–P).

To validate these results, we carried out western blots for MyHC-IIb protein levels, which were significantly reduced in the gastrocnemius and TA of A53T mice quantified by densitometry (Fig. 2Q, R, T). MyHC-IIa protein levels showed no significant change in the gastrocnemius and TA (Fig. 2Q, S, U). MyHC-slow protein levels were elevated in the gastrocnemius and soleus muscles of A53T mice by western blots and densitometry (Fig. 2V-X). Thus, these results by and large validate the immunofluorescence data with respect to the effect of PD on adult muscle fiber type, wherein MyHC-IIb protein levels are reduced, and MyHC-slow protein levels are increased in the A53T mice. The effect on MyHC-IIa protein levels is negligible in the A53T mice, with the marginal increase in MyHC-IIa+ fibers seen by immunofluorescence not observed by western blots.

To test whether the increased activity in the A53T mice causes the increase in the MyHC-slow+ fibers and decrease in the MyHC-IIb+ fibers, we subjected wild-type mice to an exercise regimen to stimulate increased muscle activity. Compared to control sedentary mice, the exercised mice showed an increase in MyHC-slow protein levels, similar to the A53T mice (Fig. S2J, K). However, unlike the A53T mice, an increase in MyHC-IIa levels and no change in MyHC-IIb levels were observed in the exercised mice (Fig. S2J-M).

These results clearly demonstrate the effect of PD on skeletal muscle fiber type. Interestingly, the strongest effects are seen on the glycolytic MyHC-IIb+ and -IIx+ fibers whose proportions are decreased, and the oxidative MyHC-slow+ fibers, which are increased in proportion. This indicates that PD leads to a gain of the oxidative MyHC-slow+ fibers at the expense of the glycolytic MyHC-IIb+ and -IIx+ fibers. This also suggests that the effect of PD may be muscle-specific, with muscles having a relatively higher proportion of glycolytic fibers affected more severely compared to those containing more oxidative fibers. Further, while physical activity could be partially responsible for the fiber type changes in the PD model, other factors such as neuronal inputs, cellular senescence and atrophy signals may also contribute to the spectrum of fiber type changes observed.

### A53T mice exhibit altered satellite cell numbers and increased fibrosis

**Fig. 3:**
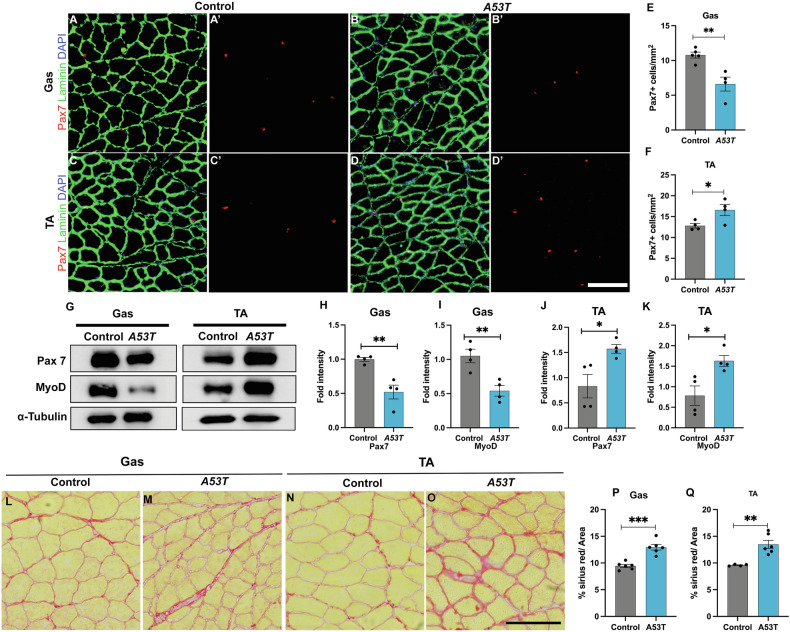
A53T mice exhibit alterations in satellite cell numbers and increased fibrosis. **A**–**F** Representative fluorescent micrographs of transverse sections through the gastrocnemius (Gas) and tibialis anterior (TA) muscle of 16 months old non-transgenic (Control) and human α-Syn hemizygous (*A53T*^+/^^−^) mice labeled for Pax7 (red), Laminin (green) and DAPI (blue) (**A**–**D')**, and quantification of the number of Pax7+ cells per unit area (mm^2^) (**E**, **F**). **G**–**K** Representative western blots for Pax7, MyoD, and α-tubulin using protein lysates from the gastrocnemius (Gas) and TA muscle of 16-month-old non-transgenic (Control) and human α-Syn hemizygous (*A53T*^+/^^−^) mice (**G**) and their densitometric quantification (**H**–**K**). **L**–**Q** Representative bright field micrographs of transverse sections through the gastrocnemius (Gas) and TA muscle of 16-month-old non-transgenic (Control) and human α-Syn hemizygous (*A53T*^+/^^−^) mice stained for Sirius red to label the extracellular matrix (**L**–**O**), and quantification of the percentage of Sirius red+ area as a fraction of total area (**P**, **Q**). Data information: Data is presented as mean ± SEM. Student’s *t* test was performed, with *p* ≤ 0.05 considered significant. Scale bar: 100 µm (**D'**, **O**).

We observed a decrease in the Pax7+ muscle stem cell (satellite cell) numbers per unit area in the gastrocnemius muscle of A53T mice at 16 months of age (Fig. 3A–B', E). However, satellite cell numbers were increased per unit area in the TA and soleus muscles of 16 months old A53T mice (Figs. 3C-D', –F and S3A–C). No change in satellite cell numbers was observed in the EDL muscle in the A53T mice (Fig. S3D–F). These results were validated by western blots and densitometry for Pax7 protein levels, which showed a decrease in the gastrocnemius and an increase in the TA in the A53T mice (Fig. 3G, H, J). Protein levels for the myogenic differentiation marker MyoD also exhibited a similar trend as Pax7, with reduced levels in the gastrocnemius and elevated levels in the TA, in the A53T mice (Fig. 3G, I, K). We observed a significant increase in the connective tissue area (marked by red in Sirius red staining), a marker of fibrosis, in cross sections through the gastrocnemius, TA, soleus and EDL muscles in the 16-month-old A53T mice (Figs. 3L–Q and S3G–J). These results further confirm that some of the effects of PD are muscle-specific, while others are broad, affecting most muscles. The effect of PD on satellite cell numbers seems to be muscle-specific, with some muscles exhibiting a decrease, others an increase, and some not affected significantly. Similar effects are observed with respect to markers of myogenic differentiation such as MyoD. However, all muscles studied showed elevated fibrosis in the PD mouse model, indicating that this effect is most likely non-muscle-specific.

### NMJ fragmentation and degeneration are seen in A53T mice

**Fig. 4:**
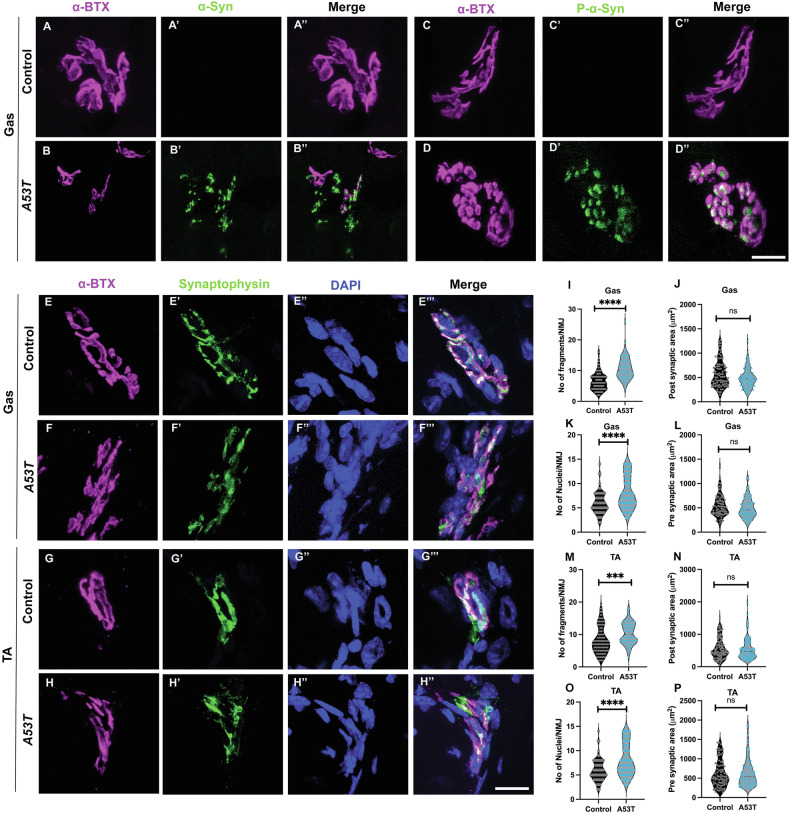
A53T mice exhibit neuromuscular junction fragmentation and degeneration. **A**–**B”** Representative fluorescent micrographs of longitudinal sections through the gastrocnemius (Gas) muscle of 16-month-old non-transgenic (Control) and human α-Syn hemizygous (*A53T*^+/^^−^) mice labeled for α-Bungarotoxin (pink), and α-Syn (green). **C**–**D”** Representative fluorescent micrographs of longitudinal sections through the gastrocnemius muscle of 16-month-old non-transgenic (Control) and human α-Syn hemizygous (*A53T*^+/^^−^) mice labeled for α-Bungarotoxin (pink), and Phospho-α-Syn (green). **E**–**P** Representative fluorescent micrographs of longitudinal sections through the gastrocnemius (Gas) and TA muscle of 16 months old non-transgenic (Control) and human α-Syn hemizygous (*A53T*^+/^^−^) mice labeled for α-Bungarotoxin (pink), Synaptophysin (green) and DAPI (blue) (**E**–**H**”), and quantification of the number of fragments/neuromuscular junction (NMJ), Post-synaptic area, Number of nuclei/NMJ and Pre-synaptic area (**I**–**P**). Data information: Data is presented as mean ± SEM. Student’s *t* test was performed, with *p* ≤ 0.05 considered significant. Scale bar: 10 µm (**D**”, **H**”).

Since we observed muscle defects and fiber type changes in the A53T mice, we next investigated the NMJs in these mice. In longitudinal sections through the gastrocnemius muscle, we found that α-Syn was expressed in the NMJs marked by α-Bungarotoxin, only in the 16-month-old A53T mice and not in controls (Fig. 4A-B”). The pathogenic Phospho-α-Syn was also expressed in the NMJs of A53T mice and not in controls (Fig. 4C-D”). These results validate the western blots for α-Syn and Phospho-α-Syn, where these were found to be expressed only in the A53T mice and not in controls (Fig. S1V, W). Further, the expression of α-Syn and Phospho-α-Syn was found to be restricted to the NMJs and not seen in other parts of the muscle in the A53T mice (data not shown). Similar results were seen with respect to α-Syn and Phospho-α-Syn expression in the NMJs in the TA muscle in A53T mice (Fig. S4A–D”). Isolated myofibers derived from enzymatically digesting stored muscle samples also exhibited α-Syn and Phospho-α-Syn expression, suggesting primary muscle defects in the A53T mice (Fig. S4E–G).

We performed immunofluorescence labeling of the presynaptic and postsynaptic areas of the NMJs using Synaptophysin and α-Bungarotoxin (labeling acetylcholine receptors), respectively, to decipher any abnormalities seen in the A53T mice. No significant difference was observed in the presynaptic or postsynaptic area occupied by NMJs in the gastrocnemius muscle of 16-month-old A53T mice (Fig. 4E-F”’, J, L). However, increased NMJ fragmentation was seen in the gastrocnemius muscle of the A53T mice (Fig. 4E-F”’, I). Interestingly, an increased number of nuclei were also seen in the NMJs of the A53T mice compared to controls (Fig. 4E-F”’, K). An increased number of nuclei in NMJs has been reported in pathological conditions such as Duchenne Muscular Dystrophy, where these help in maintaining NMJ function [35]. The results observed in the gastrocnemius muscle were validated in the TA muscle, where identical effects were seen with respect to the presynaptic and postsynaptic area, NMJ fragmentation and nuclear numbers (Fig. 4G–P). To assess whether the observed muscle defects were due to denervation, the expression of Neural Cell Adhesion Molecule 1 (NCAM1) was assessed as a marker of denervation [36]. No change in NCAM1 levels was observed between the A53T and control muscle, suggesting that denervation was not the cause of the observed muscle defects (Fig. S4H, I). These results confirm that the accumulation of α-Syn at the NMJs leads to an increase in NMJ fragmentation and nuclear number, resulting in muscle abnormalities in the A53T mice.

### A53T mice exhibit increased skeletal muscle-associated cellular senescence

**Fig. 5:**
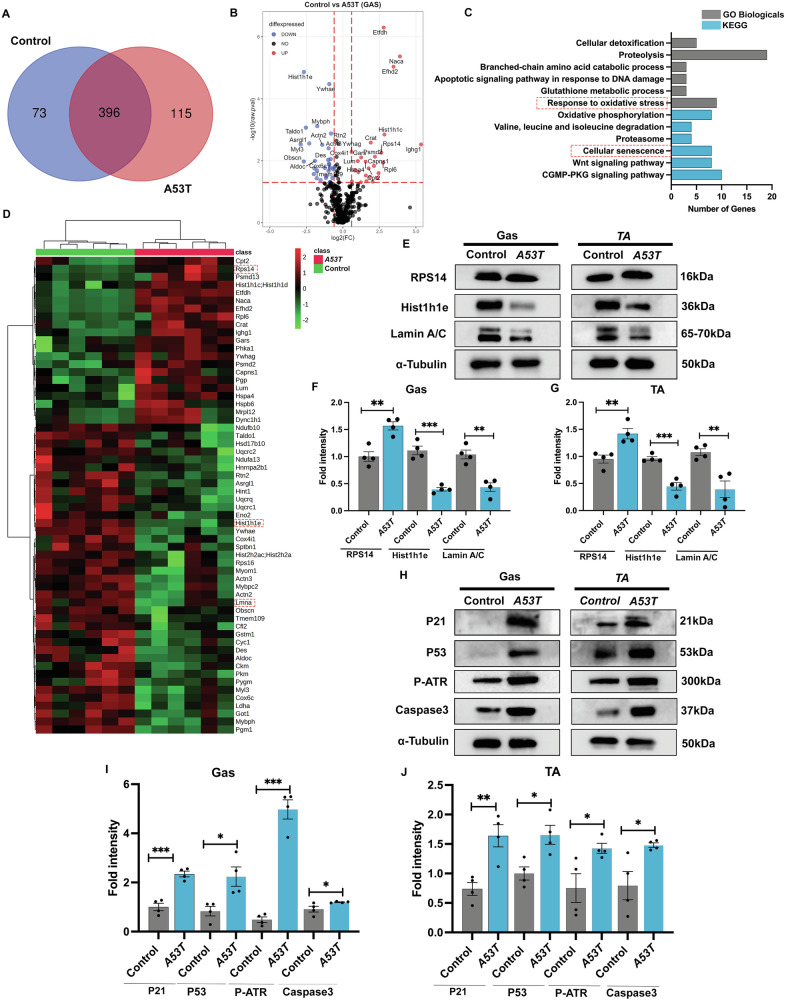
A53T mice muscle exhibits increased cellular senescence. **A** Venn diagram depicting the number of unique and shared proteins by mass spectrometry, expressed in the gastrocnemius muscle of 16-month-old non-transgenic (Control) and human α-Syn hemizygous (*A53T*^+/^^−^) mice (*n* = 6 mice per genotype). **B** Volcano plot of gastrocnemius muscle protein lysates from non-transgenic (Control) and human α-Syn hemizygous (*A53T*^+/^^−^) mice at 16 months of age, showing proteins that are differentially expressed by mass spectrometry. **C** Pathway analysis of gastrocnemius muscle protein lysates of non-transgenic (Control) and human α-Syn hemizygous (*A53T*^+/^^−^) mice at 16 months of age using GO biologicals (gray) and KEGG (blue) databases. **D** Heat map of top 50 differentially regulated proteins in the gastrocnemius muscle of non-transgenic (Control) and human α-Syn hemizygous (*A53T*^+/^^−^) mice at 16 months of age, by mass spectrometry. **E**–**G** Representative western blots for RPS14, Hist1h1e, Lamin A/C and α-tubulin using protein lysates from gastrocnemius (Gas) and tibialis anterior (TA) muscle of non-transgenic (Control) and human α-Syn hemizygous (*A53T*^+/^^−^) mice at 16 months of age (**E**), and their densitometric quantification (**F**, **G**) (*n* = 4 mice per genotype). **H**–**J** Representative western blots for P21, P53, P-ATR, Caspase-3 and α-tubulin using protein lysates from gastrocnemius (Gas) and TA muscle of non-transgenic (Control) and human α-Syn hemizygous (*A53T*^+/^^−^) mice at 16 months of age (**H**), and their densitometric quantification (**I**, **J**) (*n* = 4 mice per genotype). Data information: Data is presented as mean ± SEM. Student’s *t* test was performed, with *p* ≤ 0.05 considered significant.

To decipher the proteomic changes in the skeletal muscle in the PD mouse model, we carried out a mass spectrometric analysis of the gastrocnemius, TA, and soleus muscles of 16-month-old A53T and control mice. In the gastrocnemius, 115 proteins were found to be uniquely expressed in A53T, 73 in control, and 396 common to both genotypes (Fig. 5A). Of the 396 proteins expressed in both control and A53T gastrocnemius, 30 were downregulated and 25 upregulated in the A53T mice (Fig. 5B). In the TA muscle, 182 proteins were uniquely expressed in A53T, 49 in control, and 354 were common to both genotypes (Fig. S5A). Of the 354 common proteins expressed in control and A53T TA muscle, 19 were downregulated and 30 upregulated in the A53T mice (Fig. S5I). Similarly, in the soleus muscle, 88 proteins were uniquely expressed in A53T, 76 in control, and 377 were common to both genotypes (Fig. S5C). Of the 377 common proteins expressed in control and A53T soleus muscle, 19 were downregulated and 17 upregulated in the A53T mice (Fig. S5J). Principal component analysis (PCA) plot of the three muscles clearly showed a distinct segregation between control and A53T mice muscle proteins, where each dot depicts a biological replicate (Fig. S5 B, D, K). In all three muscles, α-Syn (SNCA) was uniquely present only in A53T mice, confirming the over-expression of α-Syn in the skeletal muscle (Supplementary Table S2). To identify the signaling pathways that are dysregulated in the skeletal muscle of the A53T mice, the proteins uniquely expressed in the gastrocnemius, TA and soleus of A53T mice were subjected to pathway analysis using the GO biologicals and KEGG pathway databases. Among several pathways that were identified, we found pathways related to oxidative stress and cellular senescence to be significantly enriched in both gastrocnemius and TA muscles (Figs. 5C and S5G, H). Other pathways that showed consistent dysregulation across muscles were proteolysis/proteasome and oxidative phosphorylation (Figs. 5C and S5G, H). Interestingly, cellular detoxification, glutathione metabolism and xenobiotic metabolic processes were dysregulated, which are also related to oxidative stress pathways (Figs. 5C and S5G, H). The top 50 differentially regulated proteins in the gastrocnemius, TA and soleus are represented in heat maps (Figs. 5D and S5E, F). We observed three interesting proteins that were associated with cellular senescence in the gastrocnemius, namely, Lamin A/C, Hist1h1e and RPS14 (Fig. 5D). While the levels of Lamin A/C and Hist1h1e were downregulated in the A53T muscle, RPS14 levels were elevated (highlighted by red box) (Fig. 5D). Reduction in levels of the nuclear envelope protein Lamin A/C has been reported to lead to increased cellular senescence [37]. Also, Lamin A/C gene mutations are associated with laminopathies, known to cause severe muscle-specific defects [38, 39]. Similarly, a mutation in Hist1h1e has been reported to lead to increased senescence and aberrant Lamin A/C morphology [40]. RPS14 is a ribosomal protein that, upon overexpression, induces senescence in cells [41]. Thus, reduced levels of Lamin A/C and Hist1h1e, and increased expression of RPS14 strongly indicate a cellular senescence response in the skeletal muscle of A53T mice.

To validate the mass spectrometry results, western blotting and densitometry were carried out for Rps14, Hist1h1e and Lamin A/C using protein lysates from the gastrocnemius and TA muscle of 16-month-old A53T and control mice. A significant increase in Rps14 levels and a decrease in Lamin A/C and Hist1h1e levels were seen in the A53T mice in both the muscles, confirming the mass spectrometry results (Fig. 5E–G). To investigate this further, we analyzed the levels of p21 and p53, well-known senescence markers whose overexpression leads to cell cycle arrest, by western blots, which were found to be significantly elevated in the gastrocnemius and TA of 16-month-old A53T mice (Fig. 5H–J). One of the mechanisms proposed for reduced Lamin A/C levels is mediated in a P-ATR-dependent manner upon DNA damage [42]. P-ATR is an important element of the DNA damage repair pathway, with increased P-ATR levels resulting from DNA damage [43]. P-ATR has been shown to phosphorylate Lamin A/C, leading to its degradation [42]. Since we find that Lamin A/C levels are decreased in the muscle of A53T mice, we checked the levels of P-ATR and found it to be significantly increased in the gastrocnemius and TA of A53T mice (Fig. 5H–J). Thus, the reduction in Lamin A/C levels in the A53T muscle is most likely due to the increased P-ATR levels, which in turn could be due to DNA damage response. The elevated phospho-ATR levels could be an indicator of cellular senescence, since ATR signaling has been reported to activate senescence in various contexts [44, 45]. Caspase-3 levels were also elevated in the gastrocnemius and TA of 16-month-old A53T mice, indicating increased apoptosis (Fig. 5H–J). Overall, these results indicate that the A53T mice exhibit increased skeletal muscle-associated cellular senescence.

### Elevated skeletal muscle oxidative stress and DNA damage are shown by A53T mice

**Fig. 6:**
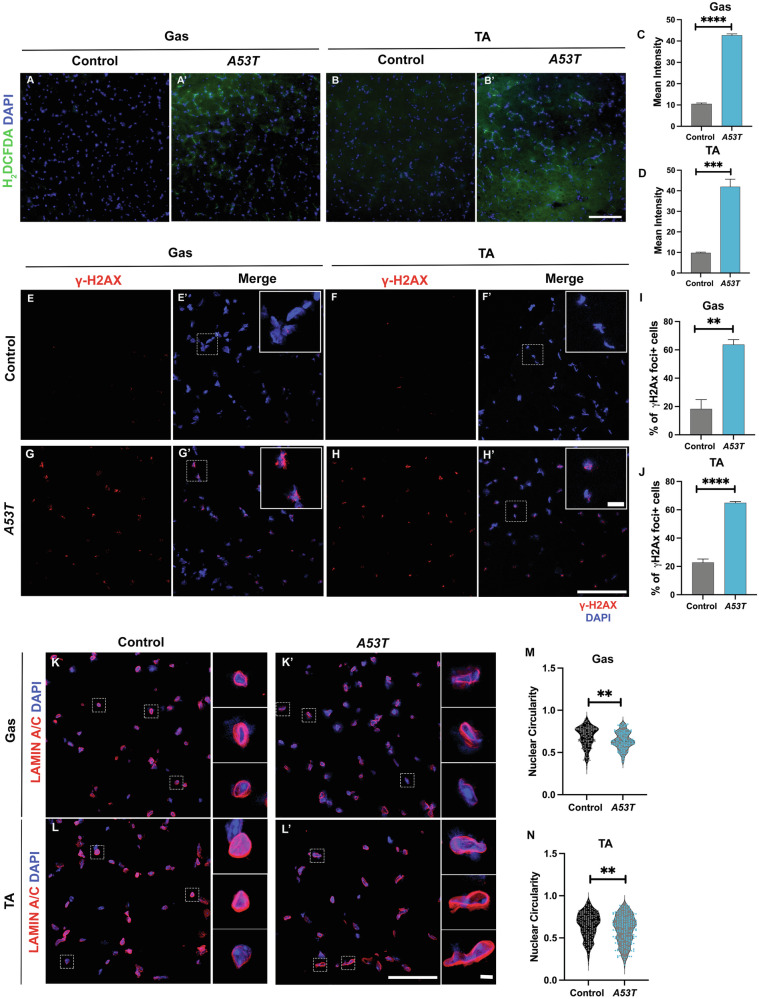
A53T mice muscle exhibits elevated oxidative stress and DNA damage. **A**–**D** Representative fluorescent micrographs of transverse sections through the gastrocnemius (Gas) and tibialis anterior (TA) muscle of 16 months old non-transgenic (Control) and human α-Syn hemizygous (*A53T*^+/^^−^) mice stained with H_2_DCFDA (green) and DAPI (blue) (**A** **-B’**), and quantification of H_2_DCFDA mean fluorescence intensity (**C**, **D**) (*n* = 3 mice per genotype). **E**–**J** Representative fluorescent micrographs of transverse sections through the gastrocnemius (Gas) and TA muscle of 16-month-old non-transgenic (Control) and human α-Syn hemizygous (*A53T*^+/^^−^) mice labeled for γ-H2AX (red) and DAPI (blue) (**E**–**H**’), and quantification of the percentage of γ-H2AX foci+ cells (**I**, **J**) (*n* = 3 mice per genotype). **K**–**N** Representative fluorescent micrographs of transverse sections through the gastrocnemius (Gas) and TA muscle of 16-month-old non-transgenic (Control) and human α-Syn hemizygous (*A53T*^+/^^−^) mice labeled for Lamin A/C (red) and DAPI (blue) (**K** **-L’**), and quantification of the nuclear circularity (**M**, **N**) (*n* = 3 mice per genotype). Data information: Data are presented as mean ± SEM. Student’s *t* test was performed, with *p* ≤ 0.05 considered significant. Scale bar: 100 µm (**B’**), 50 µm (**H’**, **L’**), 25 µm (insets of **H’** and **L’**).

Since signaling associated with oxidative stress was identified in the pathway analysis from the mass spectrometry in A53T mouse muscle, oxidative stress levels were investigated by labeling for reactive oxygen species using the dye 2′,7′-dichlorodihydrofluorescein diacetate (H2DCFDA). An increase in H2DCFDA was seen in cross sections through the gastrocnemius and TA of A53T mice compared to controls; this increase was found to be significant upon quantification of the mean fluorescence intensity (Fig. 6A-D). This validates the mass spectrometry data, confirming that A53T mice exhibit elevated oxidative stress in the skeletal muscle. Increased oxidative stress can lead to DNA damage, and we already saw an increase in levels of p53, suggesting DNA damage and the DNA damage repair pathway gene P-ATR in the skeletal muscle of A53T mice (Fig. 5H–J). To assess DNA damage, immunofluorescence labeling was done for γ-H2AX on cross-sections through the gastrocnemius and TA muscle of control and A53T mice at 16 months of age, where a significant increase in the percentage of γ-H2AX foci+ cells was seen in both muscles in the A53T mice (Fig. 6E–J).

Increased physical activity could lead to elevated oxidative stress and DNA damage. Since the A53T mice exhibit increased activity, we subjected wild-type mice to an exercise regimen to stimulate increased muscle activity. Compared to control sedentary mice, the exercised mice showed reduced levels of Cleaved Caspase-3 (reduced apoptosis) and γ-H2AX (reduced DNA damage), with no change in p21 levels, unlike the A53T mice (Fig. S6A–D). Thus, the elevated skeletal muscle apoptosis and DNA damage in the A53T mice are not due to increased activity but are most likely PD-associated muscle defects.

Lamin A/C mutations are known to affect nuclear shape and morphology [39]. Since we observed a reduction in Lamin A/C levels, we looked at the myonuclear shape of A53T mice muscle by labeling for Lamin A/C in the gastrocnemius and TA muscle. Interestingly, we observed myonuclei with distorted envelope morphology, which were quantified by an index for nuclear circularity, where a significant reduction in nuclear circularity was apparent in the myonuclei of the 16-month-old A53T mice (Fig. 6K–N). Thus, these results confirm elevated oxidative stress, increased DNA damage, and compromised nuclear circularity in the A53T mice muscle. Lamin A/C, being a nuclear envelope protein, protects the nucleus and DNA, preventing DNA damage [38]. However, a reduction in levels of Lamin A/C possibly results in reduced nuclear stability, exposing the DNA to the outer environment, making it prone to damage. Reduced Lamin A/C levels and DNA damage are closely associated [38, 46]. Another possibility is that, since skeletal muscle is a mechanically active tissue, the reduced Lamin A/C levels could result in a muscle-specific increase in DNA damage during mechanical activity, in the A53T mice [38, 39]. Thus, our results confirm that the A53T mice exhibit increased DNA damage and reduced nuclear stability, resulting in increased skeletal muscle cellular senescence.

### A53T mice exhibit SASP and enhanced muscle proteolysis

**Fig. 7:**
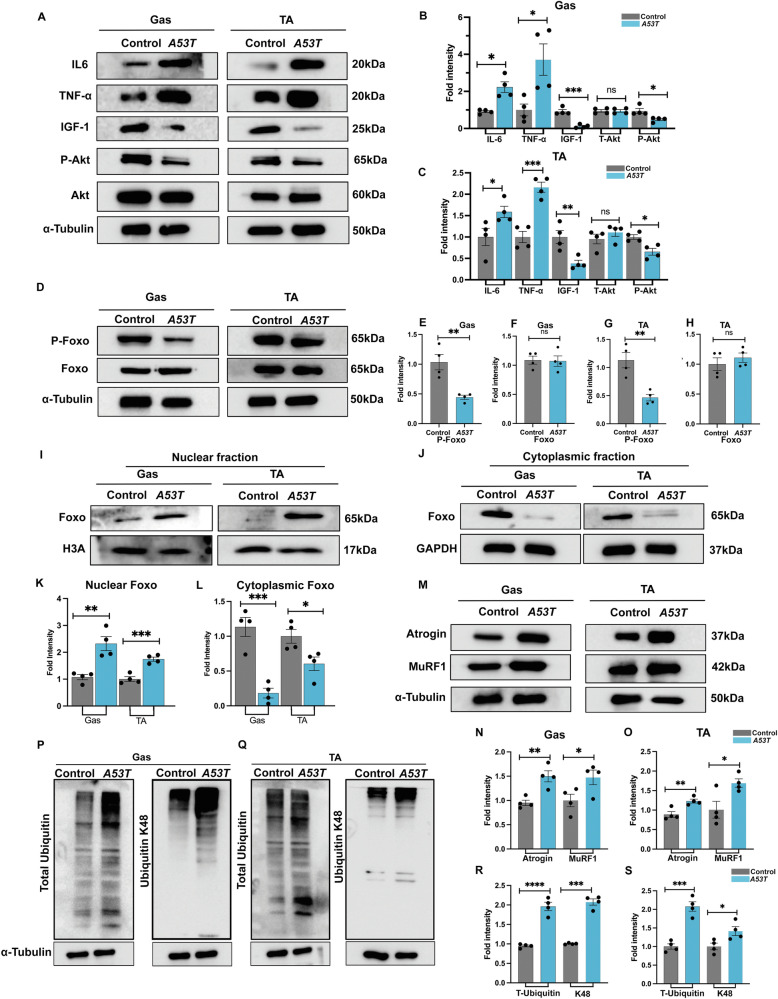
A53T mice muscle exhibits senescence-associated secretory phenotype, elevated FoxO signaling and enhanced proteolysis. **A**–**C** Representative western blots for IL6, TNF-α, IGF-1, P-Akt, total-Akt and α-tubulin, using protein lysates from gastrocnemius (Gas) and tibialis anterior (TA) muscle of non-transgenic (Control) and human α-Syn hemizygous (*A53T*^+/^^−^) mice at 16 months of age (**A**) and their densitometric quantification (**B**, **C**). **D**–**H** Representative western blots for P-FoxO, total-FoxO, and α-tubulin, using protein lysates from gastrocnemius (Gas) and TA muscle of non-transgenic (Control) and human α-Syn hemizygous (*A53T*^+/^^−^) mice at 16 months of age (**D**) and their densitometric quantification (**E**–**H**). **I**–**L** Representative western blots for total-FoxO and histone H3A in the nuclear fraction (**I**), total-FoxO and GAPDH in the cytoplasmic fraction (**J**), using protein lysates from gastrocnemius (Gas) and TA muscle of non-transgenic (Control) and human α-Syn hemizygous (*A53T*^+/^^−^) mice at 16 months of age and their densitometric quantification (**K**, **L**). **M**–**O** Representative western blots for Atrogin, MuRF1 and α-tubulin using protein lysates from gastrocnemius (Gas) and TA of non-transgenic (Control) and human α-Syn hemizygous (*A53T*^+/^^−^) mice at 16 months of age (**M**), and their densitometric quantification (**N**, **O**). **P**–**S** Representative western blots for total-Ubiquitin, K48-Ubiquitin and α-Tubulin using protein lysates from gastrocnemius (Gas) and TA of non-transgenic (Control) and human α-Syn hemizygous (*A53T*^+/^^−^) mice at 16 months of age (**P**, **Q**) and their densitometric quantification (**R**, **S**). Data information: data are presented as mean ± SEM. Student’s *t* test was performed, with *p* ≤ 0.05 considered significant.

Since we observed increased levels of senescence markers such as RPS14, p21 and p53 in the skeletal muscle of A53T mice, it is likely that there is an accumulation of senescent cells in their muscle (Fig. 5E–J). However, it is unclear whether and how these accumulated senescent cells contribute to the observed muscle mass loss in the A53T mice. Previous studies indicate that senescent cells secrete SASP molecules, mainly pro-inflammatory cytokines such as TNF-α, IL-6, etc. [18]. SASPs have been reported to inhibit or reduce the levels of IGF-1, a major determinant of muscle mass through the Akt-FoxO signaling pathway [47, 48]. Therefore, we tested whether SASPs and IGF-1 mediate the loss of muscle mass and associated phenotypes in the A53T mice. A significant increase in the protein levels of the SASPs TNF-α and IL-6, and a decrease in IGF-1 levels were observed in the gastrocnemius and TA muscle of A53T mice (Fig. 7A–C). One of the pathways leading from IGF-1 and its receptor, IGF-1R, in the skeletal muscle is mediated by Akt [49]. Therefore, we checked phospho-Akt and total-Akt levels; upon quantification, a significant reduction in the ratio of phospho-Akt normalized to total Akt levels was found in the muscle of A53T mice (Fig. 7A–C). This confirms that the reduced IGF-1 levels lead to a decrease in downstream signaling in the skeletal muscle of A53T mice.

Next, we investigated FoxO signaling due to two reasons: first, FoxO proteins are key transcription factors that regulate muscle mass and are known to be involved in muscle atrophy; second, phosphorylated-Akt has been shown to directly phosphorylate FoxO proteins to control their function [50]. For instance, serine 256 phosphorylation of FoxO1 has been reported to inactivate its function by preventing its nuclear localization or promoting its degradation [51]. We found that total FoxO1 protein levels were unchanged between control and A53T muscle, but serine 256 phospho-FoxO1 levels were significantly decreased to about half in the gastrocnemius and TA muscle of A53T mice (Fig. 7D–H). The reduced levels of phospho-FoxO1 in the A53T mice muscle indicate upregulation of the FoxO signaling pathway, which has been shown to lead to protein degradation. Since FoxO localization is dependent on its phosphorylation, to confirm the above results, we isolated the nuclear and cytoplasmic fractions from the gastrocnemius and TA muscle of 16-month-old control and A53T mice, which were then subjected to western blotting for Foxo1, H3A as a nuclear marker and GAPDH as a cytoplasmic marker (Fig. 7I–L). The nuclear fraction exhibited a significant, approximately twofold increase in FoxO1 levels, whereas the cytoplasmic fraction showed reduced FoxO1 levels in the A53T mice muscle samples, substantiating that the reduced levels of phosphorylated FoxO1 in the A53T mice indeed resulted in increased nuclear accumulation of FoxO1 and upregulation of the FoxO signaling pathway (Fig. 7I–L). Accordingly, the protein levels of two FoxO pathway target genes, muscle-specific RING finger protein 1 (MuRF1) and Atrogin, which are muscle-specific E3 ubiquitin ligases, were upregulated in the gastrocnemius and TA muscle of A53T mice (Fig. 7M–O).

A balance between protein synthesis and degradation is required to maintain skeletal muscle mass [33]. Shifting this equilibrium in either direction leads to muscle hypertrophy or atrophy. Since the FoxO signaling pathway and its targets, such as MuRF1 and Atrogin E3 ubiquitin ligases, known to ubiquitinate muscle-specific proteins, are upregulated in the muscle of A53T mice, we checked the ubiquitination levels in these mice. Elevated levels of total ubiquitination by ~two-fold were found in both the gastrocnemius and TA in the A53T mice (Fig. 7P–S). We also checked the levels of lysine 48 (K48) ubiquitination, a posttranslational modification that targets proteins for degradation by the proteasome [52]. We found that the ratio of K48 to total ubiquitin was significantly increased in the gastrocnemius and TA in the A53T mice (Fig. 7P–S). Thus, our results indicate that senescent cells secrete SASPs, which activate the FoxO signaling pathway, leading to increased expression of the FoxO pathway target genes MuRF1 and Atrogin, which are muscle-specific E3 ubiquitin ligases. This in turn results in increased protein ubiquitination and proteasomal degradation, leading to enhanced muscle proteolysis and atrophy in the muscle of A53T mice.

## Discussion

Aging in humans is associated with progressive sarcopenia or loss of skeletal muscle mass and function [53]. Sarcopenia-associated muscle dysfunction leads to impaired posture, movement, locomotor capability, increased incidence of falls and related muscle injury, and recovery from muscle damage, causing inability to perform routine daily activities and poorer quality of life [17, 54]. An ~30–50% increase in the incidence of sarcopenia, frailty and muscle dysfunction has been reported to occur in patients with PD, one of the most common neurodegenerative diseases [55, 56]. However, very few studies have focused on identifying the mechanistic basis of the muscle dysfunction associated with PD. Here, we have used the human α-Syn hemizygous *A53T*^+/^^−^ mice to characterize the muscle-associated effects seen in PD. As reported in PD patients, the A53T mice exhibit loss of skeletal muscle mass and atrophy, reduction in myofiber size, and reduced muscle function (Figs. 1A–O and S1A–O). Muscle atrophy and dysfunction are associated with a range of muscle diseases, for instance, in a mouse model for spondylocarpotarsal synostosis [57–60].

One of the most striking effects in the skeletal muscle of the A53T mice was the fiber type alterations, which we have characterized in detail in this study. A gain in the proportion of the slow, type I oxidative fibers at the expense of the fast, glycolytic type IIb and IIx fibers was apparent in multiple muscles, which possibly underlies many of the PD-associated phenotypes observed in the A53T mice, such as on oxygen consumption rate, fiber size differences and muscle function, as detailed below (Figs. 2 and S2). First, increased overall activity and a pronounced early morning spike in activity were observed in the A53T mice, with a similar increase seen in the RER and oxygen consumption (Fig. 1P–U and S1P–U). Alterations in dopamine levels, activity and metabolism have been reported in PD patients [61]. Changes in the dopamine D1 receptor and dopamine transporter have been suggested to lead to hyperactivity in a PD mouse model [62]. The increased proportion of slow, type I oxidative muscle fibers could drive the increased RER and oxygen consumption in the A53T mice. Second, a reduction in the proportion of larger fibers and an increase in the proportion of smaller fibers were seen in the A53T mice (Figs. 1F–M and S1F–M). The fast MyHC-IIb+ and MyHC-IIx+ myofibers also have the biggest fiber size. Reduction in both of these fast MyHC+ fibers contributes to the overall decline in bigger fiber size in all four representative muscles in the A53T mice (Figs. 1F–M and S1F–M). Increased proportion of type I or slow fibers with increased grouping, and greater heterogeneity in fast, type II fibers have been reported in PD patients [13, 16, 63–65]. Fast-to-slow MyHC transformation, along with atrophy and fiber type grouping, has been observed in the pharyngeal muscles of PD patients [66]. Third, the different MyHC isoforms have unique ATPase and contractile properties, which translate to functional differences between myofibers that express them [67]. Thus, the reduced grip strength and latency to fall in rotarod experiments in the A53T mice could be due to the altered proportions of fast and slow fiber types (Figs. 1N, O and S1N, O). Several studies indicate that reduced strength and impaired muscle force generation are seen in PD patients [68–72]. While it is difficult to delineate the exact contribution from the neuronal and muscle components in such impairments, fiber type alterations almost certainly play important roles. Interestingly, muscle fatigability has been reported not to occur in individuals with elevated PD motor symptoms [69, 72]. This could be due to the increase in the type I, slow fibers, which are known to be fatigue resistant, in patients with PD.

The effects on satellite cell numbers and Pax7 levels in the A53T mice were muscle dependent, with some muscles, such as the gastrocnemius, exhibiting a reduction, others, such as the TA and soleus, an increase in satellite cells and some, such as EDL, showing no change in satellite cell numbers (Figs. 3A–K and S3A–F). This could thus be a muscle-specific effect with distinct responses seen in different muscles, possibly based on activity levels, satellite cell contribution to myofiber homeostasis and severity of neuromuscular impairment. Interestingly, unlike satellite cell numbers, elevated fibrosis was uniformly seen across muscles in the A53T mice (Figs. 3L–Q and S3G–J). Satellite cells have been shown to contribute to adult myofiber homeostasis in the absence of injury, and depletion of satellite cells did not hasten or aggravate age-related sarcopenia, although it did contribute to increased fibrosis [73, 74]. PD may thus have effects on the satellite cells, possibly on their quiescence and differentiation characteristics, depending on the muscle type; however, it is unclear if reduced satellite cell function contributes to the muscle defects seen in the A53T mice. The reduced satellite cell numbers could also have effects on the skeletal muscle regenerative response in the A53T mice.

The NMJ-associated defects, such as fragmentation and an increased number of nuclei, most likely result from the accumulation of α-Syn in the NMJs of the A53T mice (Figs. 4 and S4). It is interesting that we find α-Syn and Phospho-α-Syn expression to be specific to NMJs and not in other areas within the myofibers, raising the possibility of transport of α-Syn from the brain to the NMJs; intramuscular injection of α-Syn has been previously reported to induce central nervous system disease pathology, indicating that transport of α-Syn can occur [75]. We found that Lamin A/C protein levels were downregulated to approximately half that of controls in the A53T mouse muscle, which could contribute to the observed NMJ defects (Fig. 5D–G). Interestingly, previous studies indicate the requirement of Lamin A/C in the muscle in NMJ maintenance and function [76, 77].

Cellular senescence-associated proteins were dysregulated in the skeletal muscle of A53T mice, indicating that many of the underlying muscle defects in PD might be mediated by related pathways (Figs. 5 and S5). We focused on three interesting proteins whose levels were altered in the A53T muscle, Lamin A/C, Hist1h1e and RPS14, known to play key roles in cellular senescence-associated pathways. Lamin A/C is well known for its localization to the nuclear envelope and its role in maintaining cellular homeostasis; mutations in Lamin A/C lead to premature aging syndromes such as progeria. Muscle-specific Lamin A/C knockout has been shown to lead to elevated expression of cellular senescence markers and SASPs in the muscle [37]. Hist1h1e belongs to the histone H1 family, which connects to linker DNA, aiding in chromatin compaction to regulate nucleosome structure and function. Induction of cellular senescence has been shown to lead to loss of histone H1 [78]. Interestingly, frameshift mutations in the Hist1h1e C-terminus led to increased protein stability, altered methylation patterns, reduced proliferation and aberrant cell cycle, leading to accelerated cellular senescence and aging [40]. RPS14 is a ribosomal protein that is part of the 40S ribosomal subunit. Senescent cells exhibited accumulation of RPS14 in a non-ribosomal fraction in the nucleus, causing interaction with and inhibition of cyclin-dependent kinase 4 (CDK4), leading to cell cycle arrest and senescence [41]. We also found that the classic molecular mediators of cellular senescence, such as p21, p53 and Phospho-ATR [79], were overexpressed in the skeletal muscle of A53T mice (Fig. 5H–J). ATR signaling has been shown to cause cellular senescence, generally associated with DNA damage response, and even in the absence of DNA damage [45]. Overexpression of p21 has been shown to induce cellular senescence in skeletal muscle and p53 signaling to mediate senescence during skeletal muscle regeneration [19, 80]. This suggests the accumulation of senescent cells in the skeletal muscle of A53T mice, resulting in enhanced secretion of SASPs, which we observe in the A53T muscle (Fig. 7A–C). SASPs such as IL-6 and TNF-α have been shown to inhibit IGF-1 levels in multiple contexts, including in the skeletal muscle [47, 81–83]. IGF-1 signaling regulates the muscle-specific E3 ubiquitin ligases MuRF1 and Atrogin to control skeletal muscle proteostasis through the Akt-FoxO pathway [48, 84].

We observe elevated levels of both apoptotic (Cleaved Caspase-3) and senescence-associated (p21 and p53) proteins in the skeletal muscle of the PD mouse model. This could point to the severity of damage or stresses that cells undergo, with a high degree of damage triggering apoptosis and more chronic damage leading to senescence. Another possibility is that certain cell types within the A53T mouse skeletal muscle undergo apoptosis, and others are targeted to senescence.

### Model and conclusions

**Fig. 8:**
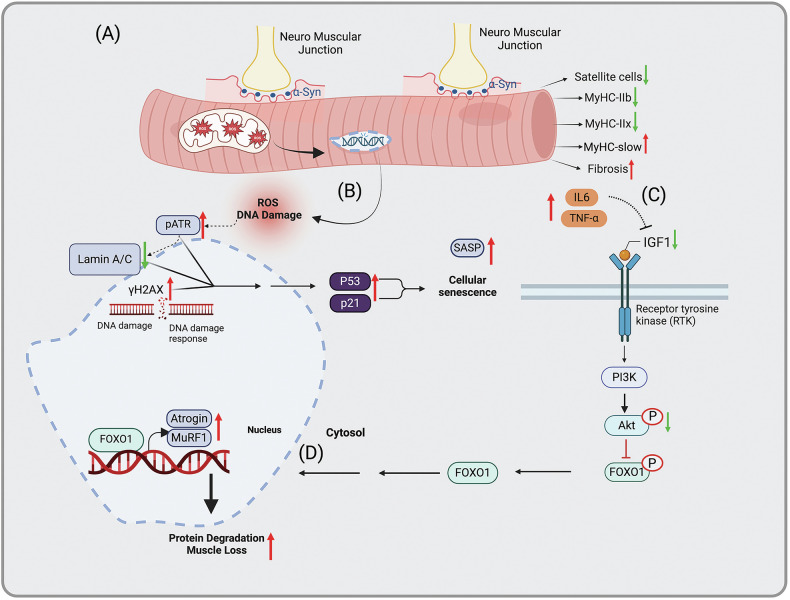
Model. **A** Accumulation of α-Syn in the neuromuscular junction (NMJ) leads to alteration in satellite cell numbers, reduction in MyHC-IIb+ and IIx+ fibers, gain of MyHC-slow+ fibers, and increased fibrosis in the myofibers. **B** Accumulation of α-Syn in the NMJs also increases reactive oxygen species (ROS) production, leading to DNA damage marked by increased levels of P-ATR and γ-H2AX. Activated P-ATR pathway leads to reduced levels of Lamin A/C, resulting in cellular senescence and enhanced secretion of senescence-associated secretory phenotype (SASP) factors such as TNF-α and IL-6. **C**, **D** Elevated levels of TNF-α and IL-6 are known to inhibit IGF-1 signaling leading to activation of the FoxO signaling cascade (**C**), resulting in upregulation of the muscle-specific E3 ubiquitin ligases Atrogin and MuRF1 (**D**). This, in turn, increases proteolysis, leading to loss of muscle mass (**D**). ‘Created with BioRender.com’.

Thus, we have characterized the skeletal muscle-specific defects using the A53T mouse model for PD, where we observe α-Syn accumulation in the NMJs, leading to NMJ defects and a range of muscle abnormalities such as gain of slow fiber type at the expense of fast fiber types, elevated fibrosis, atrophy phenotypes, altered satellite cell numbers and reduced muscle strength (Fig. 8A). Our results show that increased ROS and related DNA damage in the A53T muscle results in triggering of DNA damage response and associated pathways such as P-ATR, concomitant reduction in Lamin A/C levels and nuclear circularity, and activation of cellular senescence pathways (Fig. 8B). Senescent cells secrete SASPs, which cause a reduction in IGF-1 signaling, activation of FoxO1, leading to elevated levels of Atrogin and MuRF1 and increased protein degradation (Fig. 8C, D). Our findings support a model where accelerated aging leads to increased proteolysis and associated atrophy in skeletal muscle in PD. Thus, antioxidant and senolytic drugs could be of importance as potential therapeutics to treat the musculoskeletal defects associated with PD.

## Materials and methods

### Mice

Human α-Syn hemizygous (strain no. 010799) mice were purchased from the Jackson Laboratory, USA [34]. Genotype was determined as per protocol number 29674 (Jackson Laboratory, USA). Human α-Syn hemizygous (*A53T*^+/^^−^) and littermate non-transgenic (control) mice were selected for further experiments. Mice were euthanized at 16 months, and the tibialis anterior (TA), gastrocnemius, EDL and soleus hind limb muscles were harvested. Mice were maintained, and all animal experiments were performed according to the protocols approved by the institutional animal ethics committee (protocol number: RCB/IAEC/2022/141). Fore and hind limb grip measurements were made using a grip strength meter (GT3, Panlab, Harvard Apparatus), and values were normalized to body weight. To test the motor coordination of the control and *A53T*^+/^^−^ mice, a rotarod test was performed on a standard accelerating rotarod (Harvard Apparatus). Mice were trained on the rotarod for 2 days, and on the third day, the latency to fall was measured in seconds for the respective genotypes. To measure the volume of oxygen consumption (VO_2_) and locomotor activity, mice were allowed to acclimatize for 24 hours in a respiratory chamber. VO_2_ and locomotor activity were measured for the next 48 hours maintaining 12 hours light and 12 hours dark cycle using the Comprehensive Laboratory Animal Monitoring System (CLAMS) (Columbus Instruments). To simulate the increased muscle activity, 4-month-old wild-type C57BL/6 mice were run on a treadmill at eight meters/minute speed for 50–60 minutes, 5 days a week for 2 months on a rodent treadmill apparatus (Columbus Instruments). After 2 months of this exercise regime, the mice were euthanized at 6 months of age, and the gastrocnemius muscle from the hind limbs was harvested.

### Ex vivo contractility measurement

The muscle contractile property of non-transgenic (control) and human α-Syn hemizygous (*A53T*^+/^^−^) mice were measured at 16-months of age using a muscle contractility measurement apparatus (iWORX, IX-TA-220 data recorder and FT-302 force transducer) as reported previously [85]. Briefly, the control and A53T mice were anesthetized, and the TA muscle was partially excised by removing it from the distal tendon while keeping the proximal tendon intact. The isolated TA muscle was attached to a force transducer and a recording system to assess its contractile properties. Electrical pulses ranging from 25 Hz to 300 Hz were applied using a pair of electrodes, and the resulting force–frequency relationship was recorded. The generated forces were plotted as absolute force and maximum force (in grams).

### Immunofluorescence, histology, microscopy and image analysis

The mouse hind limb muscle tissue samples were preserved by embedding them in cryomatrix (Polysciences; 19636-1) cryo-sectioning media, rapidly frozen in chilled 2-methyl butane (Sigma; M32631) and stored at −80 °C. Sections of 10 µm thickness were obtained using a cryomicrotome (SLEE MNT), with adjacent sections collected on charged glass slides (VWR; 631-0108). Sections were cut at 30 µm thickness for samples to detect NMJs and 4 µm thickness for samples to detect lamin A/C. Slides were processed to detect MyHC isoforms, NMJs, γH2AX and oxidative stress.

For adult MyHC isoform labeling, sections were initially blocked with 10% goat serum (BioAbChem, 72-0480) and 1% Tergitol solution (Sigma; NP-40) in ice-cold phosphate-buffered saline (PBS) following acetone fixation (MyHC-IIx) or no fixation (MyHC-IIa, -IIb). Sections were then incubated overnight at 4 °C with a cocktail of isoform-specific sera and laminin antibodies in appropriate dilutions in 10% goat serum-PBS. Slides were washed first with ice-cold PBS followed by 0.5% PBS-Triton X-100, and incubated with biotin-conjugated secondary antibody (for adult MyHCs) overnight at 4 °C. This was followed by a 2-hour incubation with streptavidin-coupled fluorophore (for adult MyHCs) or fluorophore-coupled secondary antibody (for laminin) at 4 °C. Sections were rinsed in PBS, stained with Hoechst (ThermoFisher Scientific; H3570), fixed with 4% paraformaldehyde (PFA), and mounted using Fluoromount-G (Southern Biotech; 0100-01). For MyHC-slow and Pax7 stainings, sections were fixed with 4% PFA and antigen retrieval was performed using an antigen retriever (2100 PickCell Retriever; Aptum Biologics) in citrate buffer (1.8 mM citric acid and 8.2 mM sodium citrate in water). Sections were blocked with TNB blocking buffer (Perkin Elmer; FP1012) and incubated with MyHC-slow antibody or Pax7 sera diluted in TNB buffer. Signal amplification was achieved using Vectastain (Vector Laboratories; PK-6100) and TSA reagent kits (Akoya Biosciences; NEL701A001KT, NEL704A001KT). Imaging was done using an Olympus fluorescence microscope (Olympus BX63F) equipped with a Hamamatsu ORCA-Flash 4.0 camera. Maximum projections of each image were obtained, and the number of MyHC+ fibers and fiber area were quantified using SMASH (Semi-automatic muscle analysis using segmentation of histology software) [86]. CSA was measured using the CellSens Dimension 1.16 software.

To stain NMJs, α-bungarotoxin and synaptophysin were used; adjacent slides were fixed in 2% PFA at 4 °C for 2 hours, rinsed with PBS, followed by 5% goat serum blocking for 1 hour. Sections were incubated overnight with α-bungarotoxin in PBS at 4 °C. Sections were then washed thrice in PBS for 5 minutes each; slides were incubated in synaptophysin antibody at 4 °C overnight. This was followed by a 2-hour incubation with Alexa 488 fluorophore secondary antibody (for synaptophysin antibody) at room temperature. Sections were rinsed in PBS, stained with Hoechst, fixed with 4% PFA, and mounted using Fluoromount-G. For α-Syn and phospho-α-Syn, sections were fixed in 4% PFA for 5 minutes at room temperature, blocked in 5% goat serum for 1 hour and incubated overnight in primary antibody (α-Syn and phospho-α-Syn) at 4 °C. Sections were washed thrice in PBS for 5 minutes each, incubated in secondary antibodies for 2 hours at room temperature. Sections were rinsed in PBS, fixed with 4% PFA, and mounted using Fluoromount-G. To stain γH2AX and lamin A/C, sections were fixed in 4% PFA for 5 minutes at room temperature, blocked for 1 hour in 5% goat serum, followed by overnight incubation in the primary antibody (γH2AX or lamin A/C) at 4 °C. Slides were washed thrice in PBS, followed by a 2-hour incubation with Cy3-coupled secondary antibody at room temperature. Sections were rinsed in PBS, stained with Hoechst, fixed with 4% PFA, and mounted using Fluoromount-G. Imaging was done using a confocal microscope (Leica TCS SP8). For NMJs, the threshold was determined using the Huang method in Image J [87, 88]. The images were despeckled, and the pre-synaptic and post-synaptic areas were quantified. To count the number of fragments per NMJ, we set the threshold using the Huang method, applied the watershed, and used the analyze particle plugin for counting [87]. The circularity of nuclei was obtained using the circularity plugin in ImageJ [89]. γH2AX + foci were counted in ImageJ. Centrally nucleated myofibers and the density of nuclei were counted using ImageJ. Centrally nucleated myofibers were normalized to the number of myofibers, and nuclear density was normalized to the CSA. A list of antibodies used is given in Supplementary Table S1.

To visualize oxidative stress, tissue sections were stained with 2, 7′-dichlorodihydrofluorescein diacetate (H_2_DCFDA) dye. Slides were kept at room temperature for 5 minutes, followed by 30 minutes of incubation with H_2_DCFDA dye at 37 °C. Imaging was done using an Olympus fluorescence microscope (Olympus BX63F) equipped with a Hamamatsu ORCA-Flash 4.0 camera. H_2_DCFDA mean intensity was measured using ImageJ.

To investigate fibrosis, muscle tissue sections were fixed in Bouin’s fixative (75% saturated picric acid, 25% formaldehyde, and 5% glacial acetic acid) for 1 hour at 56 °C. After fixation, the sections were rinsed in tap water and stained with Picro-Sirius red (Sigma; 365548) for 1 hour. After staining, the sections were washed with 0.5% acetic acid solution. Subsequently, the stained sections were dehydrated through an ethanol series of increasing concentrations (50%, 70%, and 100%), followed by equilibration in xylene. Finally, the sections were mounted using DPX mounting medium (Sigma; 06522). The stained sections were imaged using a Nikon Eclipse Ti microscope equipped with a DS-Fi2 camera.

### Western blotting

Hindlimb muscle tissues were harvested from mice of the respective genotypes and homogenized in radioimmunoprecipitation assay (RIPA) buffer (Sigma; R0278-500ml) containing protease/phosphatase inhibitors, which were centrifuged at 13,000 g for 30 minutes. The supernatant was collected in a fresh tube, followed by quantification by BCA assay (Thermo Scientific; 23225). An equal amount of protein was loaded and separated on SDS-PAGE, followed by western blotting, where proteins were transferred to a PVDF membrane. The membrane was then blocked using 5% BSA or skimmed milk at room temperature for 1.5 hours. Blots were incubated in primary antibodies overnight at 4 °C and subsequently with HRP-conjugated secondary antibodies at room temperature for 2 hours, followed by HRP substrate detection (Millipore; WBLUF0100). The blots were imaged on an ImageQuant LAS 4000 (GE), and densitometric quantification was performed using ImageJ, where protein levels were normalized to α-tubulin levels.

To investigate the levels of α-Syn and Phospho-α-Syn in the myofibers, the quadriceps muscle was digested in collagenase for 2 hours and the digested myofibers were isolated and used for protein isolation in RIPA buffer containing protease and phosphatase inhibitors. To isolate nuclear and cytoplasmic fractions, hind limb muscle samples were harvested from mice of the respective genotypes and flash-frozen in liquid nitrogen. Samples were washed with cold PBS and homogenized in STM buffer (250 mM sucrose, 50 mM Tris-HCl pH 7.4, 5 mM Mgcl_2_) containing protease/phosphatase inhibitors. Homogenates were vortexed, followed by centrifugation at 8000 × *g* for 15 minutes. The pellet was processed for nuclear protein isolation, and the supernatant (*S*_0_) was processed for the cytoplasmic fraction. Pellets containing nuclear proteins were resuspended in STM buffer and again centrifuged at 5000 × *g* for 15 minutes. Obtained pellets were resuspended in NET buffer (20 mM HEPES pH 7.9, 1.5 mM Mgcl_2_, 0.2 mM EDTA, 20% glycerol and 1% triton x-100) containing protease and phosphatase inhibitors. To rupture the nuclei, resuspended lysates were sonicated, followed by centrifugation at 9000 × *g* for 30 minutes, and the supernatant was taken as the nuclear fraction. Cytoplasmic fraction supernatant (*S*_0_) was centrifuged at 800 × *g* for 10 minutes. The obtained supernatant was precipitated in chilled acetone at −20 °C for one hour, centrifuged at 12,000 × *g* for 5 minutes, and the pellet was dissolved in STM buffer and taken as the cytoplasmic fraction. As mentioned above, isolated nuclear and cytoplasmic fractions were estimated, and western blots were performed. H3A and GAPDH were used to normalize the quantity of proteins in the nuclear and cytoplasmic fractions, respectively [58]. A list of antibodies used is given in Supplementary Table S1. Full-length western blot images are provided as a separate Supplemental Material file.

### Mass spectrometry

Hind limb muscle tissues were harvested from mice of the respective genotypes and homogenized in RIPA buffer containing protease/phosphatase inhibitors, which were centrifuged at 13,000 × *g* for 30 minutes. The supernatant was collected in a fresh micro-centrifuge tube, followed by quantification by BCA assay. 1 milligram protein was taken for mass spectrometry sample preparation; quantified protein was precipitated in chilled acetone and kept at −20 °C for 1 hour, followed by overnight incubation at −80 °C. Samples were centrifuged at 6000 × *g* for 45 minutes at 4 °C, followed by two washes in acetone. The obtained pellet was dissolved in 8 M urea, centrifuged at 10,000 × *g* for 10 minutes, the supernatant was taken and quantified by BCA assay, and 100 µg of protein was processed further. Briefly, after adding Dithiothreitol (Sigma; 53819), the samples were incubated for 1 hour at 560 °C, which was followed by the addition of cysteine blocking agent (IAA) (Sigma; 15161) and incubated in the dark at room temperature for 1 hour. Samples were then digested with trypsin (1:20, Thermo Scientific; 90057) at 37 °C for 18 hours. Subsequently, the tryptic peptides were dried using a speed vacuum concentrator at 25 °C. The dried samples were desalted using Pierce C18 Zip-tips (Thermo Scientific; 87782) and again vacuum dried and reconstituted in buffer A (2% acetonitrile, 0.1% formic acid) for further LC-MS/MS analysis.

Each sample was run in triplicate on a Triple TOF 5600+ (Sciex, Concord, Canada) equipped with an Eksigent NanoLC-2DPlus system (Eksigent, Dublin, CA, USA) consisting of a Chrom-XP reverse-phase 3 μm C18-CL trap column (350 μm × 0.5 mm, 120 Å, Eksigent) and nanoViper C18 separation column (75 μm × 250 mm, 3 μm, 100 Å; Acclaim Pep Map, Thermo Scientific).

MaxQuant software (v1.6, Max Planck Institute of Biochemistry, Germany) was utilized to identify and quantify peptides and proteins. Intensity data from raw files were used for additional analysis and comparisons amongst the groups. Statistical analysis was carried out using the Metaboanalyst web tool (v5).

Two-sided unpaired Student’s *t* tests were used to compare the groups statistically. First, statistical significance was determined using the threshold *p* value of <0.05. Then, the statistically significantly differentially expressed genes were used to enrich pathways. ShinyGO (v7.6) tool was employed to perform over-representation analysis using the KEGG and GO biologicals database for pathway enrichment. The PCAs and heatmap visualization were done using Metaboanalyst. Other graphical representations were made using the ggplot2 package (v3.5.1) in R (v4.3.1) program. The mass spectrometry data are given in Supplementary Table S.

### Statistical analysis

The experimental data were analyzed utilizing GraphPad Prism 9 software (GraphPad Software Inc., CA, USA) and presented as mean ± standard error of the mean (SEM). Statistical analysis employed a parametric, two-tailed, unpaired *t* test to assess the probability of significant differences, with significance defined as *P* values ≤ 0.05 (*). No statistical methods were used to pre-determine sample sizes; however, the number of samples used is comparable to those reported in a previous study [58]. Animal studies were conducted with at least three biological replicates for each experimental condition, with sample number details provided in the figures and legends. Experimental conditions, stimulus presentation, and animal studies were not randomized, with data collection and analyses conducted without blinding.

## Supplementary information

Supplementary Data

Supplementary Table 2

Uncropped Blots

## Data Availability

All data needed to evaluate the conclusions in the paper are present in the paper/Supplementary Information.
