# Supplementary material for: LaminA/C-dependent cellular senescence signaling promotes skeletal muscle atrophy and abnormalities in Parkinson’s disease
Source: Cell Death Dis. 2026 Jun 6;17(1):709. doi: 10.1038/s41419-026-08962-0 (PMC13463094; doi:10.1038/s41419-026-08962-0)
Supplement: Supplementary file 1 [file 41419_2026_8962_MOESM1_ESM.pdf]

Supplementary Information

LaminA/C-dependent cellular senescence signaling promotes skeletal muscle atrophy and abnormalities in Parkinson's Disease.

Jaydeep Sharma[1], Somnath Mondal[1], Krishna Singh Bisht[2], Ankit Biswas[2], Lakshmikanthan Panneerselvam[1], Tushar Kanti Maiti[2], and Sam J. Mathew[1,#]

[#] - Corresponding Author: Sam J. Mathew (ORCiD: 0000-0001-7908-6094)

Developmental Genetics Laboratory

Regional Centre for Biotechnology (RCB), NCR Biotech Science Cluster,

3rd Milestone, Faridabad-Gurgaon Expressway, Faridabad, 121001, Haryana, India.

Telephone: +91 129-284-8822 **Email**: sjmathew@rcb.res.in

List:

Supplementary Figures:

Figure S1. A53T mice exhibit muscle structural and functional defects.

Figure S2. A53T mice exhibit alterations in muscle fiber type.

Figure S3. A53T mice exhibit alterations in satellite cell numbers and increased fibrosis.

Figure S4. α-Syn is expressed in the neuromuscular junctions (NMJ) of *A53T* mice.

Figure S5. A53T mice muscle exhibit increased cellular senescence.

Figure S6. Exercise-trained wild type mice exhibit reduced DNA damage and apoptosis.

Supplementary Tables:

Supplementary Table S1. List of antibodies used.

Supplementary Table S2. Mass spectrometry results.

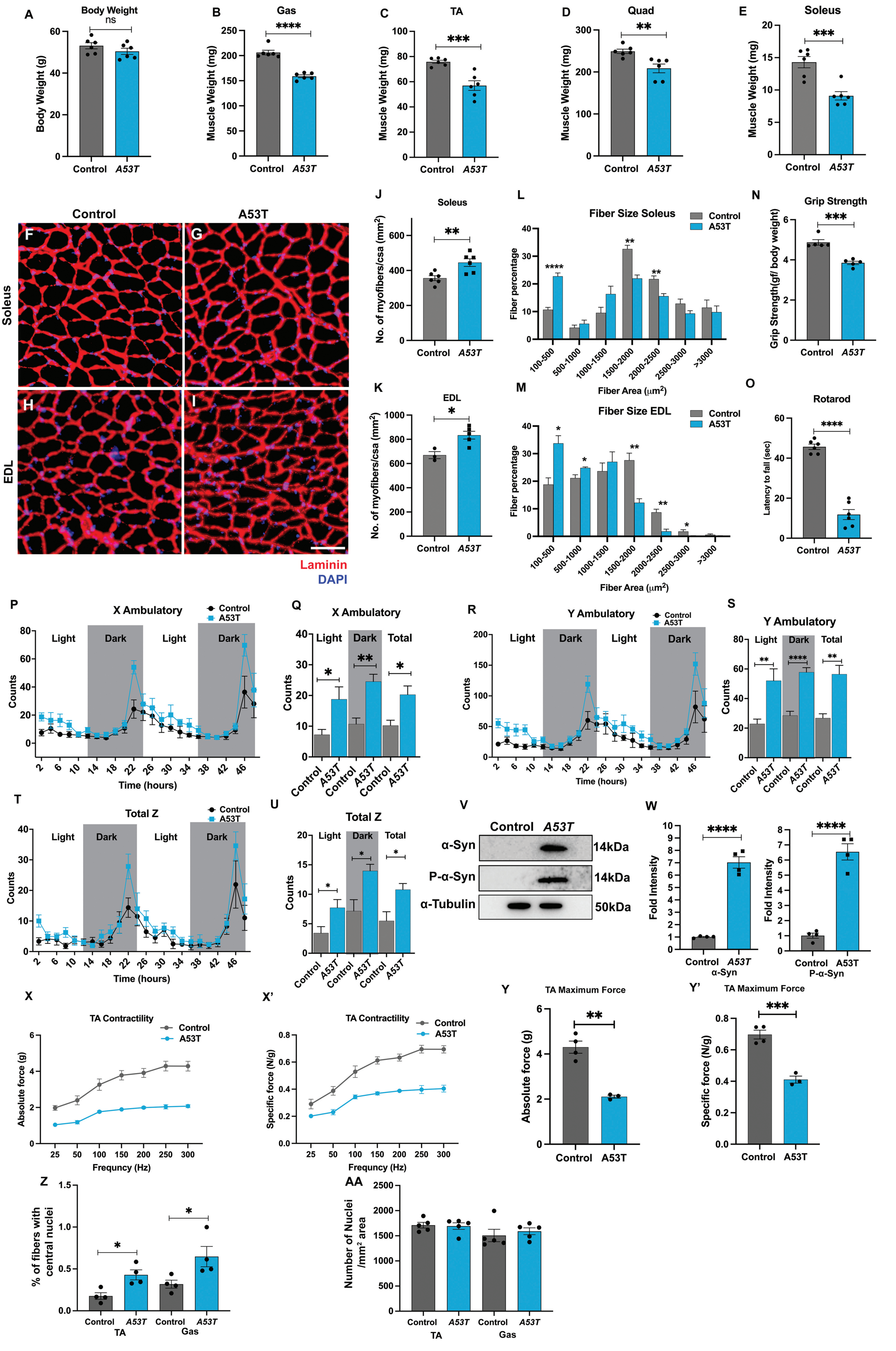
A
Body Weight
ns
Body Weight (g)
Control
A53T
B
Gas
****
Muscle Weight (mg)
C
TA
***
D
Quad
**
E
Soleus
***
Control
A53T
F
G
H
I
Soleus
EDL
Laminin
DAPI
J
Soleus
No. of myofibers/csa (mm2)
K
EDL
L
Fiber Size Soleus
Fiber percentage
Fiber Area (µm2)
100-500
500-1000
1000-1500
1500-2000
2000-2500
2500-3000
>3000
M
Fiber Size EDL
N
Grip Strength
Grip Strength(gf/ body weight)
O
Rotarod
Latency to fall (sec)
P
X Ambulatory
Light
Dark
Counts
Time (hours)
Q
X Ambulatory
Total
R
Y Ambulatory
S
Y Ambulatory
T
Total Z
U
Total Z
V
α-Syn
P-α-Syn
α-Tubulin
14kDa
14kDa
50kDa
W
Fold Intensity
α-Syn
P-α-Syn
X
TA Contractility
Absolute force (g)
Frequncy (Hz)
X'
TA Contractility
Specific force (N/g)
Frequncy (Hz)
Y
TA Maximum Force
Absolute force (g)
Y'
TA Maximum Force
Specific force (N/g)
Z
% of fibers with central nuclei
TA
Gas
AA
Number of Nuclei /mm2 area
TA
Gas

Figure S1. A53T mice exhibit muscle structural and functional defects.

A. Total body weight (grams) of non-transgenic (Control) and human α-Syn hemizygous (*A53T*$^{+/-}$) male mice measured at 16 months of age.

B-E. Individual muscle weight (milligrams) of gastrocnemius (Gas), tibialis anterior (TA), quadriceps (Quad), and soleus of non-transgenic (Control) and human α-Syn hemizygous (*A53T*$^{+/-}$) male mice measured at 16 months of age.

F-I. Representative fluorescent micrographs of transverse sections through the soleus and extensor digitorum longus (EDL) muscle of non-transgenic (Control) and human α-Syn hemizygous (*A53T*$^{+/-}$) mice measured at 16 months of age labeled for Laminin (red), and DAPI (blue).

J, K. Quantification of the number of myofibers normalized to cross-sectional area (CSA) (mm^2) through the soleus and EDL muscle of non-transgenic (Control) and human α-Syn hemizygous (*A53T*$^{+/-}$) mice measured at 16 months of age.

L, M. Quantification of the number of myofibers grouped according to myofiber area through the EDL and soleus muscles of non-transgenic (Control) and human α-Syn hemizygous (*A53T*$^{+/-}$) mice measured at 16 months of age (n=4 mice per genotype).

N. Quantification of grip strength of non-transgenic (Control) and human α-Syn hemizygous (*A53T*$^{+/-}$) male mice normalized to body weight (gf/gram body weight) at 16 months of age.

O. Rotarod analysis of 16 months old non-transgenic (Control) and human α-Syn hemizygous (*A53T*$^{+/-}$) male mice depicting the mean time of latency to fall (seconds) of individual mice.

P-U. Graphs quantifying the hourly (P, R, T) and average (Q, S, U) total locomotor activity (X, Y and Z) of 16 months old non-transgenic (Control) and human α-Syn hemizygous (*A53T*$^{+/-}$) female mice (n=6 mice per genotype).

V-W. Representative western blots for α-Syn, Phospho α-Syn and α-tubulin using protein lysates from gastrocnemius and TA of non-transgenic (Control) and human α-Syn hemizygous (*A53T*$^{+/-}$) mice at 16 months of age (V) and their densitometric quantification (W).

X-Y'. Ex-vivo contractility quantifying the absolute, specific and maximum absolute specific force normalized to muscle mass of TA muscle contractility of non-transgenic (Control) and human α-Syn hemizygous (*A53T*$^{+/-}$) mice measured at 16 months of age. (n=3-4 mice per genotype).

Z-AA. Quantification of the percentage of myofibers with centralized myonuclei (Z) and the number of nuclei/mm^2 area (AA) of TA and gastrocnemius muscles from non-transgenic (Control) and human α-Syn hemizygous (*A53T*$^{+/-}$) mice measured at 16 months of age.

Data information: Data is presented as mean ± SEM. Student's t-test was performed, with $p \leq 0.05$ considered significant. Scale bar: 100 μm (I).

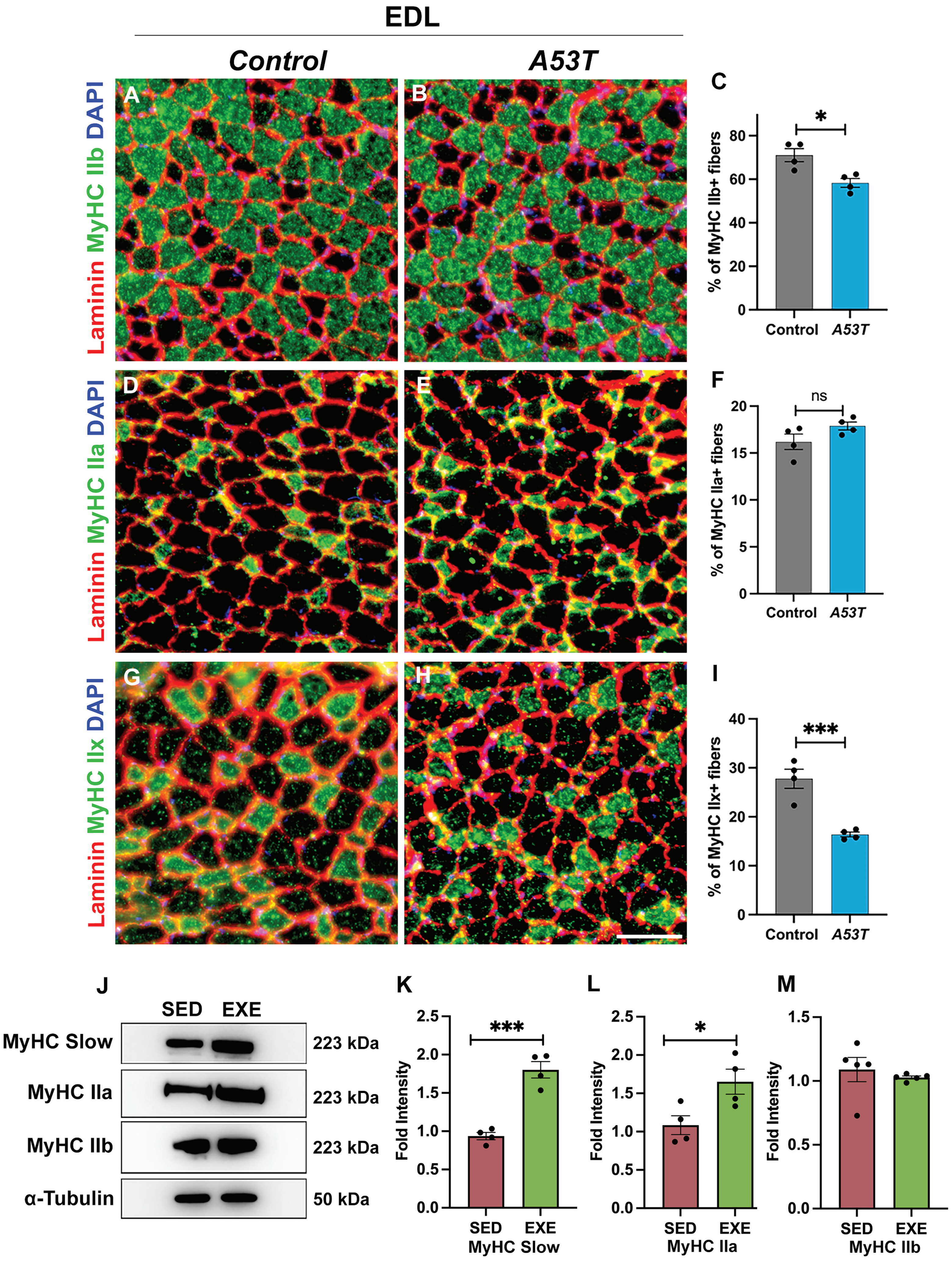

EDL
Control
A53T
Laminin MyHC IIb DAPI
Laminin MyHC IIa DAPI
Laminin MyHC IIx DAPI
A
B
C
D
E
F
G
H
I
% of MyHC IIb+ fibers
% of MyHC IIa+ fibers
% of MyHC IIx+ fibers
*
ns
***
Control
A53T
J
SED
EXE
MyHC Slow
MyHC IIa
MyHC IIb
α-Tubulin
223 kDa
223 kDa
223 kDa
50 kDa
K
L
M
Fold Intensity
*
***
SED
EXE
MyHC Slow
MyHC IIa
MyHC IIb

Figure S2. A53T mice exhibit alterations in muscle fiber type.

A-C. Representative fluorescent micrographs of transverse sections through the extensor digitorum longus (EDL) muscle of 16 months old non-transgenic (Control) and human α-Syn hemizygous ($A53T^{+/-}$) mice labeled for Laminin (red), MyHC-IIb (green) and DAPI (blue) (A-B), and quantification of the percentage of MyHC-IIb+ fibers (C).

D-F. Representative fluorescent micrographs of transverse sections through the EDL muscle of 16 months old non-transgenic (Control) and human α-Syn hemizygous ($A53T^{+/-}$) mice labeled for Laminin (red), MyHC-IIa (green) and DAPI (blue) (D-E), and quantification of the percentage of MyHC-IIa+ fibers (F).

G-I. Representative fluorescent micrographs of transverse sections through the EDL muscle of 16 months old non-transgenic (Control) and human α-Syn hemizygous ($A53T^{+/-}$) mice labeled for Laminin (red), MyHC-IIx (green) and DAPI (blue) (G-H), and quantification of the percentage of MyHC-IIx+ fibers (I).

J-M. Representative western blots for MyHC-slow, MyHC-IIa, MyHC-IIb and α-tubulin (loading control) using protein lysates from the gastrocnemius (Gas) muscle of sedentary (SED) and 2-month-exercised (EXE) wild type mice (J), and their densitometric quantification (K-M).

Data information: Quantification of MyHC+ fibers is represented as percent of MyHC+ fibers normalized to total number of fibers. Data is presented as mean ± SEM. Student's t-test was performed, with $p \leq 0.05$ considered significant. Scale bar: 100 μm (H).

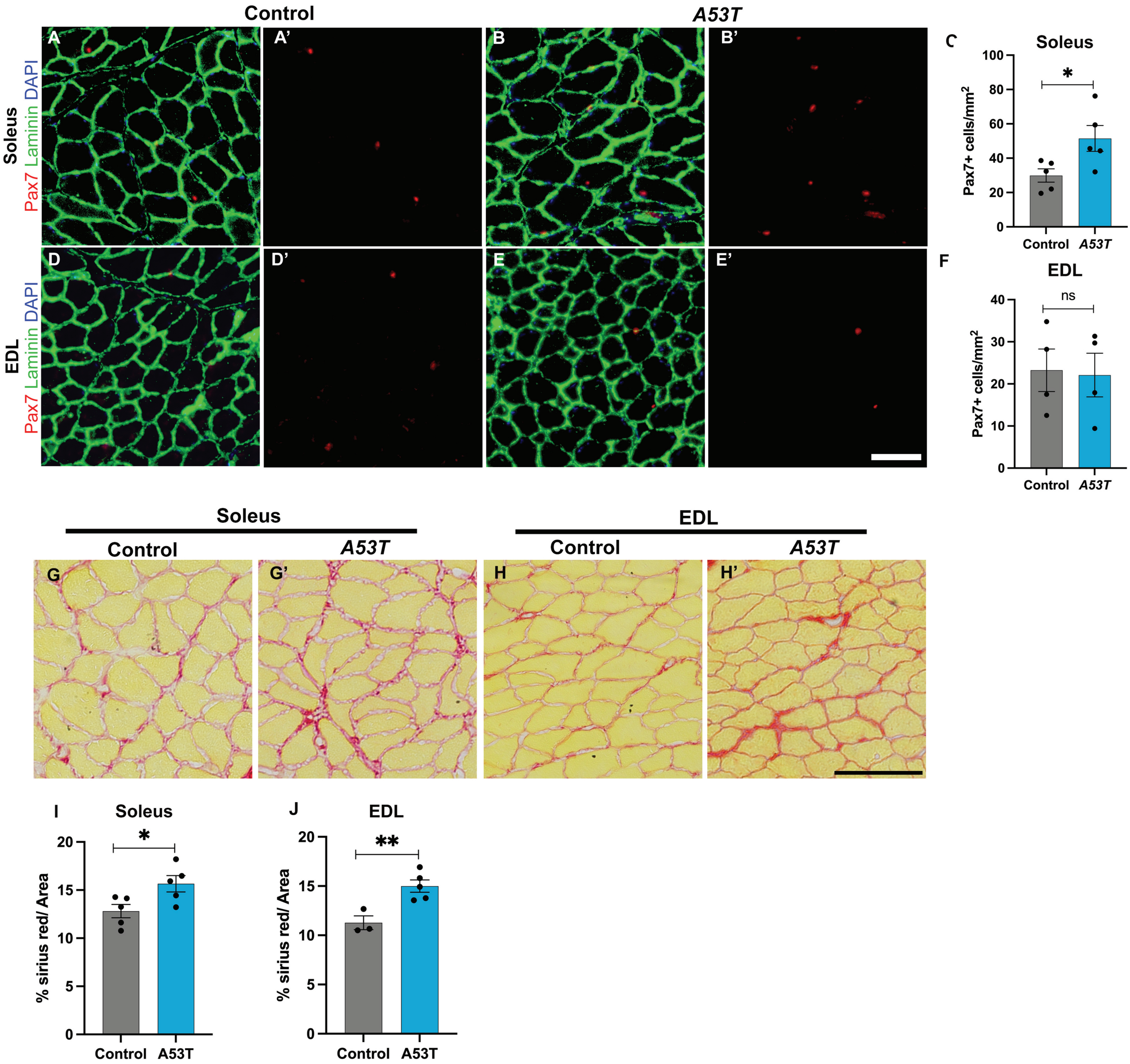

Control
A53T
A
A'
B
B'
C
Soleus
Pax7 Laminin DAPI
Pax7+ cells/mm2
100
80
60
40
20
0
Control
A53T
*
D
D'
E
E'
F
EDL
Pax7 Laminin DAPI
40
30
20
10
0
ns
Soleus
EDL
Control
A53T
G
G'
H
H'
I
Soleus
% sirius red/ Area
20
15
10
5
0
*
Control
A53T
J
EDL
**

Figure S3. A53T mice exhibit alterations in satellite cell numbers and increased fibrosis.

A-F. Representative fluorescent micrographs of transverse sections through the soleus and extensor digitorum longus (EDL) muscle of 16 months old non-transgenic (Control) and human α-Syn hemizygous ($A53T^{+/-}$) mice labeled for Pax7 (red), Laminin (green) and DAPI (blue) (A-B', D-E'), and quantification of the number of Pax7+ cells per unit area (mm^2) (C, F).

G-J. Representative bright field micrographs of transverse sections through the soleus and EDL muscle of 16 months old non-transgenic (Control) and human α-Syn hemizygous ($A53T^{+/-}$) mice stained for Sirius red to label the extracellular matrix (G-H'), and quantification of the percentage of Sirius red+ area as a fraction of total area (I, J).

Data information: Data is presented as mean ± SEM. Student's t-test was performed, with $p \leq 0.05$ considered significant. Scale bar: 100 µm (E', H').

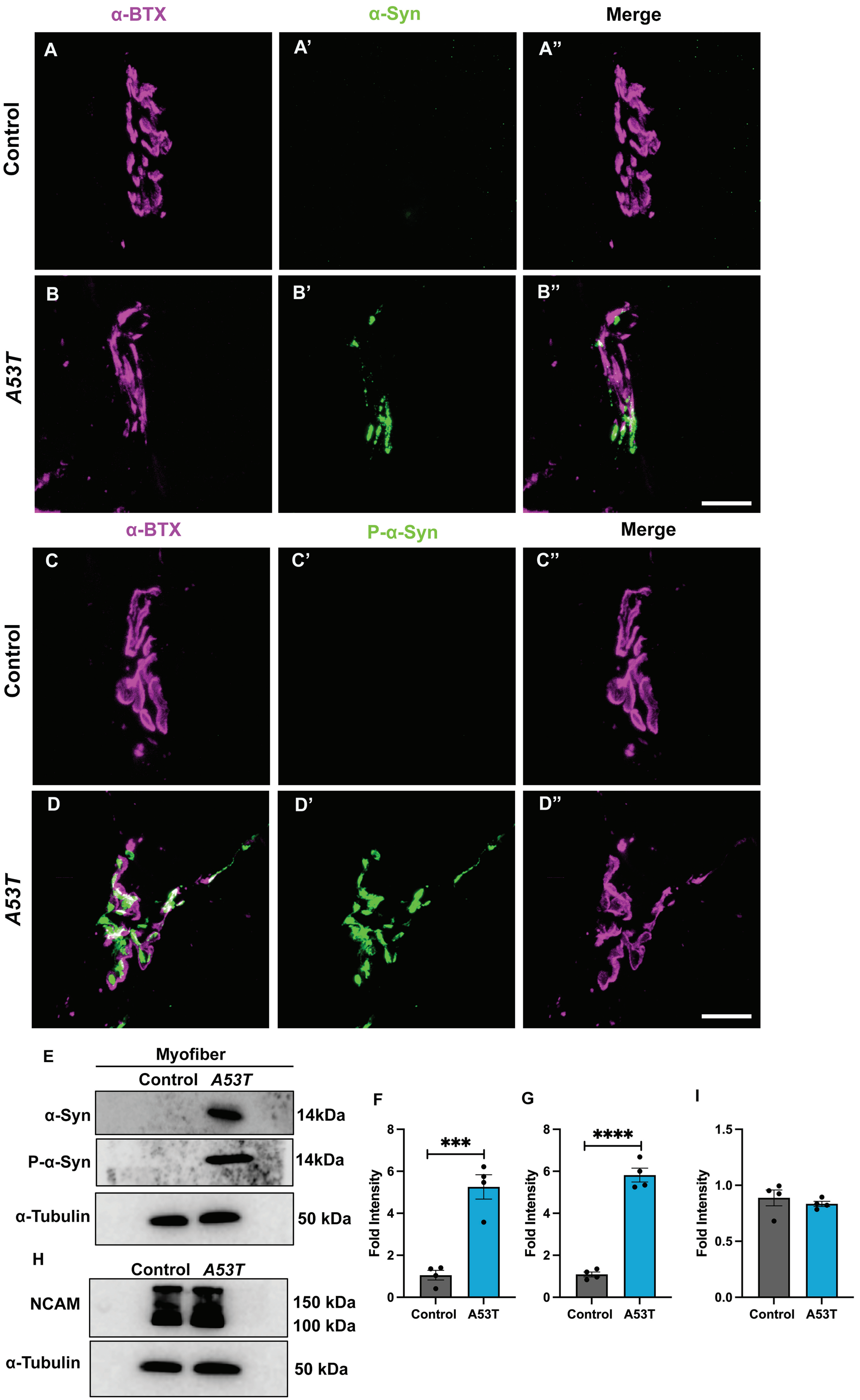

α-BTX
α-Syn
Merge
A
A'
A''
Control
B
B'
B''
A53T
α-BTX
P-α-Syn
Merge
C
C'
C''
Control
D
D'
D''
A53T
E
Myofiber
Control
A53T
α-Syn
14kDa
P-α-Syn
14kDa
α-Tubulin
50 kDa
H
Control
A53T
NCAM
150 kDa
100 kDa
α-Tubulin
50 kDa
F
G
I
Fold Intensity
***
****
Control
A53T

Figure S4. α-Syn is expressed in the neuromuscular junctions (NMJ) of *A53T* mice.

A-B''. Representative fluorescent micrographs of longitudinal sections through the tibialis anterior (TA) muscle of 16 months old non-transgenic (Control) and human α-Syn hemizygous ($A53T^{+/-}$) mice labeled for α-bungarotoxin (pink), α-Syn (green) and DAPI (blue) (A-B'').

C-D''. Representative fluorescent micrographs of longitudinal sections through the TA muscle of 16 months old non-transgenic (Control) and human α-Syn hemizygous ($A53T^{+/-}$) mice stained with α-bungarotoxin (pink), Phospho- α-Syn (green) and DAPI (blue) (C-D'').

E-G. Representative western blots for α-Syn, Phospho- α-Syn and α-tubulin (loading control) using protein lysates from isolated myofibers of quadriceps muscle of non-transgenic (Control) and human α-Syn hemizygous ($A53T^{+/-}$) mice at 16 months of age (E) and their densitometric quantification (F-G).

H-I. Representative western blots for Neural Cell Adhesion Molecule (NCAM) and α-tubulin (loading control) using protein lysates from gastrocnemius (Gas) muscle of non-transgenic (Control) and human α-Syn hemizygous ($A53T^{+/-}$) mice at 16 months of age (H) and their densitometric quantification (I).

Data information: Data is presented as mean ± SEM. Student's t-test was performed, with $p \leq 0.05$ considered significant. Scale bar: 10 μm (B'', D'').

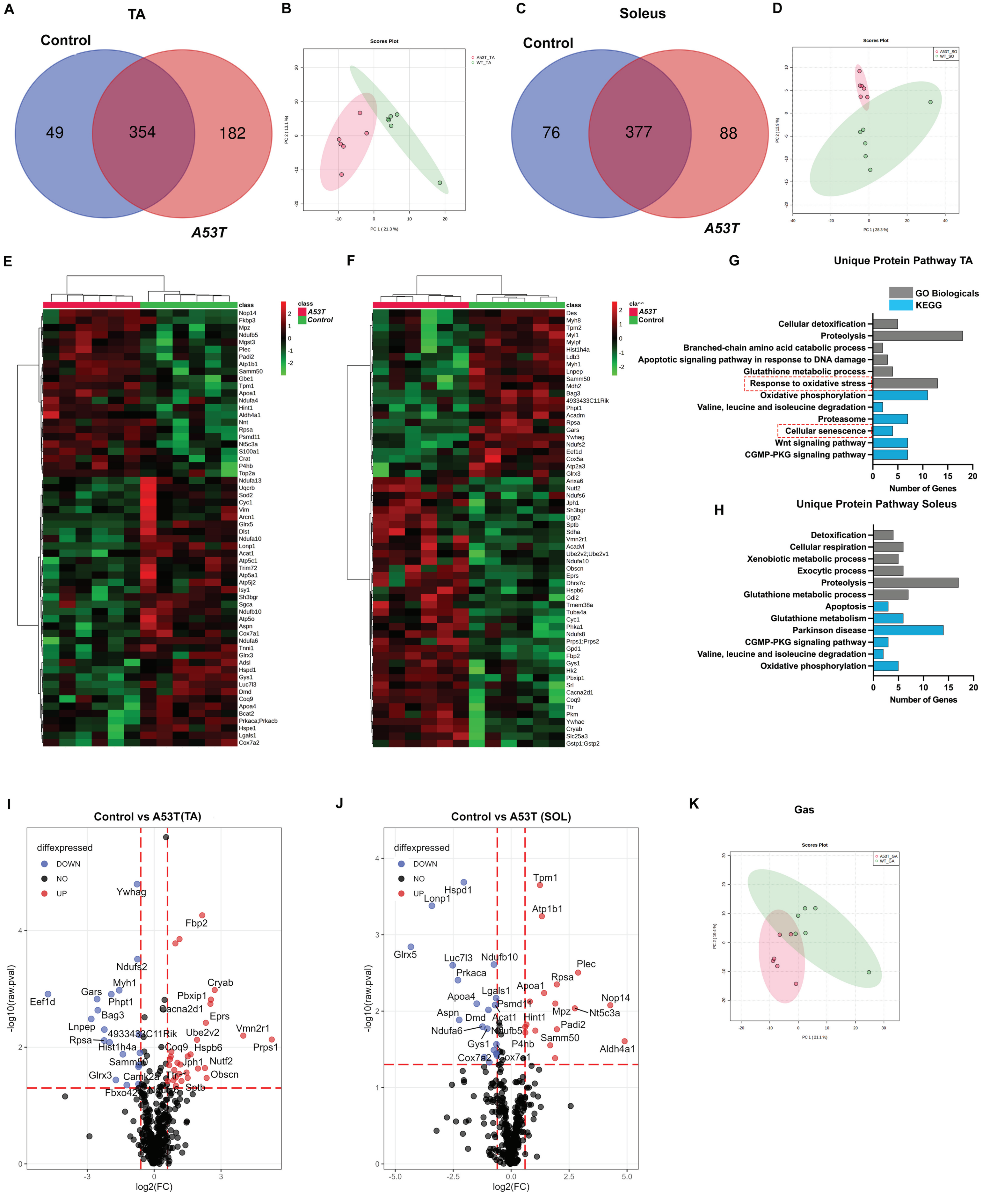

A
TA
Control
49
354
182
A53T
B
Scores Plot
C
Soleus
Control
76
377
88
A53T
D
Scores Plot
E
F
G
Unique Protein Pathway TA
GO Biologicals
KEGG
Cellular detoxification
Proteolysis
Branched-chain amino acid catabolic process
Apoptotic signaling pathway in response to DNA damage
Glutathione metabolic process
Response to oxidative stress
Oxidative phosphorylation
Valine, leucine and isoleucine degradation
Proteasome
Cellular senescence
Wnt signaling pathway
CGMP-PKG signaling pathway
Number of Genes
H
Unique Protein Pathway Soleus
Detoxification
Cellular respiration
Xenobiotic metabolic process
Exocytic process
Proteolysis
Glutathione metabolic process
Apoptosis
Glutathione metabolism
Parkinson disease
CGMP-PKG signaling pathway
Valine, leucine and isoleucine degradation
Oxidative phosphorylation
Number of Genes
I
Control vs A53T(TA)
diffexpressed
DOWN
NO
UP
-log10(raw.pval)
log2(FC)
J
Control vs A53T (SOL)
diffexpressed
DOWN
NO
UP
-log10(raw.pval)
log2(FC)
K
Gas
Scores Plot

Figure S5. ***A53T*** **mice muscle exhibit increased cellular senescence.**

A. Venn diagram depicting the number of unique and shared proteins by mass spectrometry, expressed in the tibialis anterior (TA) muscle of 16 months old non-transgenic (Control) and human α-Syn hemizygous (*A53T*$^{+/-}$) mice (n=6 mice per genotype).

B. PCA plot for TA samples of non-transgenic (Control) and human α-Syn hemizygous (*A53T*$^{+/-}$) mice at 16 months of age. Each dot represents one biological replicate, with control (green) and A53T (red).

C. Venn Diagram depicting the number of unique and shared proteins by mass spectrometry, expressed in the soleus muscle of 16 months old non-transgenic (Control) and human α-Syn hemizygous (*A53T*$^{+/-}$) mice (n=6 mice per genotype).

D. PCA plot for soleus muscle samples of non-transgenic (Control) and human α-Syn hemizygous (*A53T*$^{+/-}$) mice at 16 months of age. Each dot represents one biological replicate, with control (green) and A53T (red).

E, F. Heat map of top 50 differentially regulated proteins in the TA and soleus muscle of non-transgenic (Control) and human α-Syn hemizygous (*A53T*$^{+/-}$) mice at 16 months of age, by mass spectrometry.

G, H. Pathway analysis of TA and soleus muscle protein lysates of non-transgenic (Control) and human α-Syn hemizygous (*A53T*$^{+/-}$) mice at 16 months of age using the KEGG database.

I, J. Volcano plot of TA and soleus muscle protein lysates from non-transgenic (Control) and human α-Syn hemizygous (*A53T*$^{+/-}$) mice at 16 months of age, showing proteins that are differentially expressed by mass spectrometry.

K. PCA plot for gastrocnemius (Gas) muscle samples of non-transgenic (Control) and human α-Syn hemizygous (*A53T*$^{+/-}$) mice at 16 months of age. Each dot represents one biological replicate, with control (green) and A53T (red).

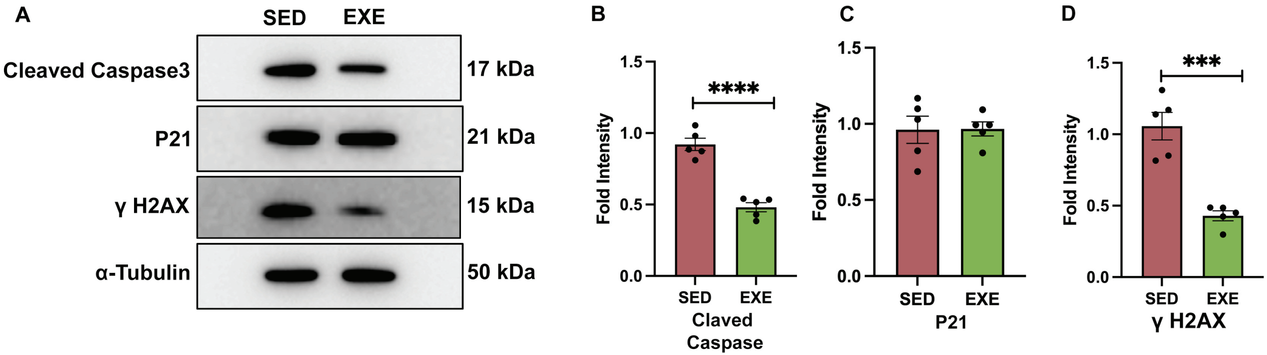

A
SED
EXE
Cleaved Caspase3
17 kDa
P21
21 kDa
γ H2AX
15 kDa
α-Tubulin
50 kDa
B
Fold Intensity
****
SED
EXE
Claved Caspase
C
Fold Intensity
SED
EXE
P21
D
Fold Intensity
***
SED
EXE
γ H2AX

Figure S6. Exercise-trained wild type mice exhibit reduced DNA damage and apoptosis.

A-D. Representative western blots for Cleaved Caspase 3, p21, γ-H2AX and α-tubulin (loading control) using protein lysates from the gastrocnemius (Gas) muscle of sedentary (SED) and 2-month-exercised (EXE) wild type mice (A) and their densitometric quantification (B-D).

Data information: Data is presented as mean ± SEM. Student's t-test was performed, with $p \leq 0.05$ considered significant.

Supplementary Table S1: Antibodies used for immunofluorescence and western blots

Antibody	Type	Source	Product No.	Working Dilution
**Primary antibodies**				
Pax7	Mouse IgG1	Developmental Studies Hybridoma Bank	PAX7	1:10 (IF, WB)
Laminin	Rabbit polyclonal	Sigma	L9393	1:400 (IF)
MyHC slow	Mouse IgG1	Sigma	M8421 (NOQ7.5.4D)	1:1000 (IF, WB)
MyHC-IIa	Mouse IgG1	Developmental Studies Hybridoma Bank	SC-71	1:10 (IF, WB)
MyHC-IIb	Mouse IgM	Developmental Studies Hybridoma Bank	BFF3	1:10 (IF, WB)
MyHC-IIx	Mouse IgM	Developmental Studies Hybridoma Bank	6H1	1:50 (IF, WB)
Alpha Tubulin	Mouse IgG	Cell Signaling Technology	30021290	1:10000 (WB)
Ubiquitin	Rabbit IgG	Cell Signaling Technology	43124S	1:2500 (WB)
K48-Linkage specific polyubiquitin	Rabbit IgG	Cell Signaling Technology	8081S	1:2500 (WB)
MuRF1	Rabbit IgG	Thermo Fisher Scientific	PA576695	1:2000 (WB)
Synaptoph ysin	Rabbit IgG	Thermo Fisher Scientific	A11043	1:500 (IF)
CDKN1A P21	Rabbit IgG	Abclonal	A2691	1:1000 (WB)
RPS14	Rabbit IgG	Abclonal	A4094	1:2000 (WB)
FBX32	Rabbit IgG	Abclonal	A3699	1:2000 (WB)
Anti-Histone H3	Rabbit IgG	Abcam	Ab1791	1:10000 (WB)
FOXO1	Rabbit IgG	Abclonal	A2934	1:2000 (WB)
P-FOXO1 (S256)	Rabbit IgG	Abclonal	AP0172	1:2000 (WB)
Lamin A/C	Rabbit IgG	Thermo Fisher Scientific	MA5-35284	1:2500 (WB) 1:800 (IF)
Histone H1.4	Rabbit IgG	Thermo Fisher Scientific	702876	1:1000 (WB)

IGF1	Rabbit IgG	Abcam	Ab223567	1:2500 (WB)
AKT	Rabbit IgG	Cell Signaling Technology	4641	1:2000 (WB)
P-AKT (S473)	Rabbit IgG	Cell Signaling Technology	4060	1:2000 (WB)
Caspase3	Rabbit IgG	Cloud-Clone Corp.	PAA636-Mu01	1:2000 (WB)
P-ATR (S428)	Rabbit IgG	Cell Signaling Technology	2853	1:2000 (WB)
MyoD	Mouse IgG	Santa-Cruz	Sc-32758	1:400 (WB)
Phospho-Histone H2A.X (S139)	Rabbit IgG	Cell Signaling Technology	9718	1:500 (IF)
GAPDH	Rabbit IgG	Cloud-Clone Corp.	PAB932Mu01	1:10,000 (WB)
IL6	Rabbit IgG	Abclonal	A2447	1:2000 (WB)
TNF-alpha	Rabbit IgG	Abclonal	A11534	1:2000 (WB)
Alpha-Syn	Rabbit IgG	Cell Signaling Technology	2642	1:2000 (WB)<br>1:500 (IF)
P-Alpha Syn (S129)	Rabbit IgG	Cell Signaling Technology	23706	1:2000 (WB)<br>1:800 (IF)
P53	Mouse IgG	Cell Signaling Technology	2524	1:2000 (WB)
NCAM	Mouse IgG	Cell Signaling Technology	123C3	1:2000 (WB)
**Secondary antibodies**				
Cy2/Cy3 conjugated Goat anti-rabbit	Goat	Jackson ImmunoResearch Laboratories	111-225-144/ 111-165-144	1:500 (IF)
Biotin conjugat edGoat anti-mouse	Goat	Jackson ImmunoResearch Laboratories	115-065-020	1:500 (IF)
Biotin conjugated Goat anti-rabbit	Goat	Jackson ImmunoResearch Laboratories	111-065-144	1:500 (IF)
AF-594 Alpha Bungarotoxin		Thermo Fisher Scientific	B13423	1:500 (IF)
Cy2 conjugated streptavidin	-	Jackson ImmunoResearch Laboratories	016-220-084	1:500 (IF)

Cy3 conjugated streptavidin	-	Jackson ImmunoResearch Laboratories	016-160-084	1:500 (IF)
Peroxidase-AffiniPure Goat anti-rabbit	Goat	Jackson ImmunoResearch Laboratories	111-035-144	1:10,000 (WB)
Peroxidase-AffiniPure Goat anti-mouse	Goat	Jackson ImmunoResearch Laboratories	111-035-003	1:10,000 (WB)
