# Supplementary material for: LaminA/C-dependent cellular senescence signaling promotes skeletal muscle atrophy and abnormalities in Parkinson’s disease
Source: Cell Death Dis. 2026 Jun 6;17(1):709. doi: 10.1038/s41419-026-08962-0 (PMC13463094; doi:10.1038/s41419-026-08962-0)
Supplement: Supplementary file 2 [file 41419_2026_8962_MOESM2_ESM.pdf]

Supplementary Table S2

Unique in Control		Unique in A53T		DEPs	FC
Dpy19l1		Gm1564		Myl3	0.13505
Hist1h2ak		Hk2		Obscn	0.15652
Cfap44		Psmb1		Hist1h1e	0.15725
Pdlim1		Sypl1		Taldo1	0.17262
Tcap		Snca		Asrgl1	0.20255
Psmc3		Vmn2r1		Sptbn1	0.25161
Tom1		C3		Hsd17b10	0.25959
Tuba1b		Tuba1b		Aldoc	0.26566
Gpi		Gpi		Hnrnpa2b1	0.28858
S100a4		Serpina3k		Lmna	0.29174
Hsp90aa1		Cbr2		Mybph	0.29575
Nefl		Fth1		Atp2a2	0.34668
Eif4a1		Calml3		Actn2	0.37537
Gstm2		Alad		Hist2h2ac;Hist2h2aa1;Hist2h2ab	0.42642
Rpl13a		Eif4a2		Ndufb10	0.44749
Serpina1b		Hist3h2ba		Rps16	0.45919
Bgn		Sptb		Des	0.498
Gnb4		Gstm2		Ndufa13	0.50652
Rpl18		Ctsd		Actn3	0.50796
Cox7a2		Serpinh1		Cox6c	0.51675
Cox8b		Cox5b		Tmem109	0.52128
Myl2		Abcb4		Ywhae	0.52562
Wdhd1		Pcmt1		Pdhb	0.54057
Rab10		Ces1c		Uqcrq	0.54175
Cops2		Rdx		Cdh13	0.54748
Ppp1cb		Pdia3		Cox4i1	0.55901
Olfm3		Adh5		Rtn2	0.57012
Ppp3ca		Gsr		Uqcrc2	0.60887
Grin2c		Fmod		Eno2	0.61983
Nme2		Myl2		Cyc1	0.65671
Ank1		Slc25a5		Ywhag	1.5124
Ckb		Cnbp		Tubb4b;Tubb4a;Tubb5;Tubb2b;Tubb2a;Tubb3	1.5375
Ndufb6		Atp5e		Pgp	1.7754
Spata5		Cox7a1		Lum	2.031
Ksr2		Rac2		Phka1	2.0812
Jakmip3		Cdc42		Ddt	2.345
Nab2		Rab10		Gars	2.3788
Ogn		Hnrnpk		Hspa4	2.9462
Uggt1		Ppp1cc		Hspb6	2.9754
Smarca1		Rps13		Rpn2	3.0663
Atp1a2		Rps3		Cpt2	3.3873
Syne1		Ppp3ca		Dync1h1	3.6057
Akap9		Cct2		Crat	3.7598
Ppp2r1a		Cct6a		Cryab	4.1121
Nap1l4		Sgca		Capns1	4.3655
Smap2		Rpl19		Psmd2	4.5681
Bzrap1		Psme1		Psmd13	5.1337
Minos1		Serpina1d		Mrpl12	5.4384
Mast4		Nme2		Rps14	6.2171
Hnrnpa3		Anxa7		Rpl6	6.7234
Rtp1		Acads		Etfdh	6.9291
Cacnb1		Cd36		Hist1h1c;Hist1h1d	7.1933
Trpm8		Jsrp1		Efhd2	11.068
Ces1d		Tgtp1		Naca	14.987
Pdcd10		Smarca4		Ighg1	40.79
Myh7		Eif3j1			
Ak5		Katnal2			

Camk2g		Elane			
Ophn1		Timm8a1			
Mlycd		Mypn			
Pgls		Psmb6			
Lgals2		Irgm1			
Psmd8		Serpinb6			
Ndufa8		Adipoq			
Pias4		A2m			
Ak2		Spr			
Tnni1		Vcl			
Noc2l		Gart			
Atp6v1c1		Mylk			
Unc13b		Ccdc57			
Fkbp1b		Atp1a2			
Gnb1		Dna2			
Gnb2		Fhod3			
		Ppp2r1a			
		Mtch2			
		Samm50			
		Ehd2			
		Dcakd			
		Psmd5			
		Stac3			
		Alms1			
		Hibch			
		Acat1			
		Aamdc			
		Cacnb1			
		Rnh1			
		Dst			
		Camk2g			
		Mpi			
		Mpst			
		Hibadh			
		Aspn			
		Rtn4			
		Ndufa5			
		Ndufa2			
		Cuta			
		Ndufb4			
		Rps21			
		Acot13			
		Dnajc19			
		Rpl14			
		Nsfl1c			
		Sdhc			
		Nt5c3a			
		Acp1			
		Ndufb8			
		Itpa			
		Smpx			
		Hrg			
		Jph1			
		Ybx3			
		Iigp1			
		Ptges3			
		Psma4			
		Camk2d			

List of Proteins in TA

Unique in Control	Unique in A53T	DEPs	FC
Tbc1d25	Ckap5	Eef1d	0.036755
Gm4477	Murc	Lnpep	0.14131
Prdx4	Casq2	Gars	0.16962
Acot1	Kpna3	Bag3	0.17315
Mtx2	Capn1	Rpsa	0.21083
Serpina1c	Mdm4	4933433C11Rik	0.21229
Myl4	Hnrnph1	Hist1h4a	0.24976
Calml3	Snca	Phpt1	0.26367
Gstm1	Atp2a2	Glrx3	0.30337
Irf1	Psmb5	Myh1	0.3368
Gstm2	Eef1b	Samm50	0.37767
Prkca	Tcap	Fbxo42	0.4263
Kif3a	Capns1	Ywhag	0.58712
Ckmt1	Tom1	Ndufs2	0.59435
Hist1h1e	C3	Camk2a	0.61452
Lum	Ighg1	Myl1	0.6147
Rab10	Ighm	Myh8	0.62122
Hspa8	Apoa4	Des	0.62415
Tubb4b	Me1	Tpm2	0.64099
Stim1	S100a4	Atp5l	1.5251
Naca	Phkg1	Ndufa10	1.5963
Hist1h2ba	Cbr2	Phka1	1.6387
Top2a	Gstm1	Gys1	1.6744
Nme2	Hist3h2ba	Tmem38a	1.7064
Spr	H1f0	Gstp1	1.8221
Hist2h2ac	Rps16	Sh3bgr	1.8706
Ppp2r1a	Hspb1	Tuba4a	1.935
Bzrap1	Rplp0	Gdi2	1.956
Fbxo5	Gstm2	Sdhc	1.9656
Csmd3	Hist1h1c	Dhrs7c	2.0588
Glrx5	Ddx3y	Hk2	2.1736
Mast4	Selenbp2	Ywhae	2.2004
Npas4	Ctsd	Ndufs8	2.2606
Cacnb1	Tpm3	Ndufs6	2.3373
Ak5	Abcb4	Ttr	2.7366
Camk2g	Gc	Sptb	2.839
Mpi	Serpina1b	Coq9	2.841
Gtpbp4	Pdia3	Hspb6	3.1036
Pccb	Gnb2	Ube2v2	3.7911
Kif19	Agtr1b	Jph1	3.9044
Ndufa5	Dbi	Fbp2	4.4557
Sar1b	Rab6a	Nutf2	4.8533
Mettl16	Rpl18	Eprs	4.9894
Itpa	Hist1h1d	Obscn	5.1122
Acadsb	Rplp1	Cacna2d1	5.7495
Pfn2	Rpl13	Pbxip1	5.8445
Tuba8	Cox7a2	Cryab	6.5747
Extl1	Cox8b	Vmn2r1	15.989
Sytl4	Ahcy	Prps1	38.763
	Fmod		
	Myl2		
	Lum		
	Cpt2		
	Atp5e		
	S100a1		
	Actb		
	Rac2		

		Rab10			
		Ube2k			
		Arf5			
		Dad1			
		Hnrnpk			
		Rps18			
		Rps4x			
		Rps6			
		Ran			
		Rap1b			
		Rps3			
		Rpl8			
		Pfn1			
		Hspa8			
		Olfm3			
		Rps10			
		Ppp3ca			
		Tubb4b			
		Tuba1c			
		Rock1			
		Naca			
		Sgca			
		Psme1			
		Rps5			
		Rplp2			
		Serpina1c			
		Gbp1			
		Nme2			
		Pros1			
		Npepps			
		Tgtp1			
		Xxylt1			
		Wdr19			
		Ndufb6			
		Gpd1l			
		Katnal2			
		Zzef1			
		Hnrnpd			
		Psmb6			
		Irgm1			
		Serpinb6			
		Psap			
		Erbb3			
		Fxr1			
		A2m			
		Ogn			
		Actr3			
		Hist2h2ac			
		Fam214a			
		Tpm4			
		Eif4g1			
		Uggt1			
		Lrpprc			
		Syne1			
		Ppp2r1a			
		Rpf1			
		Hnrnpa3			
		Psmd11			
		Fam210a			

		Psmd5			
		Trub1			
		Stbd1			
		Phf20l1			
		Rdh13			
		Akr7a2			
		Pygb			
		Crocc			
		Hibch			
		Mterf3			
		Cacnb1			
		Lrrc8c			
		Prpsap2			
		Cops8			
		Ubxn4			
		Psmd2			
		Pdcd10			
		Dnah5			
		Rnh1			
		Adcy4			
		Grhpr			
		Dars			
		Camk2g			
		Esam			
		Mpst			
		Pacsin3			
		Hibadh			
		Epdr1			
		Aspn			
		Rtn4			
		Hspb2			
		Ndufc2			
		Ndufa3			
		Sar1b			
		Ndufb9			
		Ndufb3			
		Rpl14			
		Ndufs4			
		Tecr			
		Ndufb8			
		Fundc2			
		Tm9sf1			
		Eif3f			
		Paics			
		Gstk1			
		Jph2			
		Dync1h1			
		Pdk2			
		Pias4			
		Txnrd1			
		Glrx			
		Kcnip3			
		Slc25a13			
		Iigp1			
		Fhl3			
		Bcam			
		Cdh13			
		Psmd13			
		Ndufa7			

	Fxyd1		
	Fkbp1b		
	Psma5		
	Dnpep		
	Slc25a20		
	Ighg3		
	Gstm7		

List of Proteins in Soleus

Unique in Control	Unique in A53T	DEPs	FC
Tbc1d25	Ccdc183	Glrx5	0.049837
Ryr1	Armc4	Lonp1	0.09343
Clock	Chadl	Luc7l3	0.17398
Dctn1	Bin1	Prkaca;Prkacb	0.20364
Wdr1	Prdx4	Aspn	0.21168
Vti1a	Kera	Hspd1	0.24278
Klk1b1	C1qbp	Apoa4	0.35699
Gfap	Snca	Ndufa6	0.42383
S100a4	Acot1	Coq9	0.44734
Serpina1c	Eef1b	Gys1	0.48998
Serpina3k	Gpld1	Dmd	0.50738
Anxa1	Cops6	Uqcrb	0.52125
Gstm1	Mtatp6	Cox7a2	0.59656
Col1a1	C3	Ndufb10	0.59767
Hist1h1c	Mtnd1	Acat1	0.61816
Sptan1	Mbp	Lgals1	0.63745
Selenbp2	Serpina1c	Hspe1	0.64163
Il5ra	Nefl	Cox7a1	0.6432
Serpina1b	Eif4a1	Sh3bgr	0.65631
Sar1b	Gstm1	Ndufb5	1.5196
Capza2	Hist3h2ba	P4hb	1.5279
Actg1	Tnni2	Hint1	1.5824
Rab10	Rplp0	Psmd11	1.7494
Ube2n	Sptb	Samm50	2.0596
Arf5	Hist1h1c	Tpm1	2.3662
Nutf2	Ddx3y	Atp1b1	2.509
Ppp1cc	Selenbp1	Apoa1	2.6763
Rap1b	Ctsd	Gbe1	3.2145
Tubb4b	Gc	Crat	3.7196
Cct5	Serpina1b	Mpz	3.7469
Anxa4	Ces1c	Rpsa	3.9191
Serpina1d	Rps2	Padi2	3.93
Enah	Msn	Nt5c3a	6.7235
Pdlim7	Glud1	Plec	7.4048
Gls2	Ephx2	Nop14	19.38
Aldh16a1	Flt1	Aldh4a1	29.761
Ift88	Cox8b		
Gdi2	Ahcy		
Postn	Map3k1		
Dazl	Mp68		
Vcl	Actb		
Atg2a	Rab10		
Lrpprc	Ube2k		
Ano8	Ube2l3		
Atp1a2	Tubb4b		
Zc3h4	Idh3g		
Ppp2r1a	Cct2		
Actn1	Anxa11		
Pomt2	Uba1		

Snip1		Znf488			
Synpo2l		Zdbf2			
Ttc22		Abr			
Hgh1		Gdi2			
Rtp1		Exoc3			
Ppp4r1		Mylk			
Amotl2		Erc2			
Cmbl		Ccdc57			
Clybl		Atp1a2			
Synpo2		Cand1			
Trim34a		Usp34			
Ndufc2		Akap9			
Sdhc		Cmya5			
Ube2v2		Ppp2r1a			
Ndufb8		Csmd3			
Lyzl6		Vwa8			
Prkar1a		Top1mt			
Ranbp2		Atp1a1			
Xpo4		Dst			
Dync1h1		Acaa1a			
Rpl38		Mpst			
Acot2		Hibadh			
Pclo		Epdr1			
Galk1		Ndufa5			
Cdh13		Glo1			
Timm10b		Fis1			
Unc13b		Dnajc19			
		Mrto4			
		Acp1			
		Atad1			
		Fundc2			
		Ppa1			
		Pgam1			
		Gstk1			
		Hspb8			
		Srcin1			
		Ak2			
		Pdcd6ip			
		Slc25a20			

List of Pathways

Soleus Pathway Analysis					
Pathway_GO Biologicals	Enrichment FDR	nGenes	Pathway Genes	Fold Enrichment	
Glutathione metabolic process	3.81E-05	7	70	18.86637931	Gstm7 Gstm3 Glo1 Gstm4 Gstk1 Gstm2 Gstm1
Proteolysis	0.107135921	17	1799	1.782815166	Ddx3x Uba1 Ctsd Fis1 Cops6 Gpld1 C3 Bin1 Snca Ube2k Pdcd6ip Ube2l3 Mbp Usp34 Serpina1a Serpina1b Serpina1e
Exocytic process	0.000693136	6	86	13.16259022	Rab8a Rab10 Snca Rab13 Rab8b Erc2
Xenobiotic metabolic process	0.008784692	5	102	9.248225152	Gstm7 Gstm4 Acaa1a Gstm2 Gstm1
Cellular respiration	0.021878966	6	215	5.265036087	Idh3g Ndufa5 Cox8b Snca mt-Nd1 mt-Atp6
Detoxification	0.050280996	4	123	6.135407906	Gstm7 Prdx4 Gstk1 Gstm2
KEGG					

Parkinson disease	8.39E-09	14	262	10.08127139	Tubb6 Tubb5 Uba1 Ndufa5 Cox8b Snca Tubb4b Ube2l3 Tubb2b Tubb2a Tubb3 Tubb4a mt-Nd1 mt-Atp6
Glutathione metabolism	2.46E-05	6	72	15.72198276	Gstm7 Gstm3 Gstm4 Gstk1 Gstm2 Gstm1
Oxidative phosphorylation	0.004025892	5	133	7.092623801	Ppa1 Ndufa5 Cox8b mt-Nd1 mt-Atp6
Valine, leucine and isoleucine degradation	0.100229288	2	56	6.737992611	Hibadh Acaa1a
Apoptosis	0.100229288	3	136	4.161701318	Ctsd Actb Actg1
CGMP-PKG signaling pathway	0.143273076	3	171	3.309891107	Atp1a2 Mylk Atp1a1
TA Pathway Analysis					
Pathway_GO Biologicals					
Branched-chain amino acid catabolic process	0.091408736	2	17	11.18052738	Hibadh Hibch
Regulation of intrinsic apoptotic signaling pathway in response to dna damage	0.056815083	3	36	7.91954023	Pias4 Rps3 Hnrnpk
Cellular detoxification	0.035860032	5	93	5.109380793	Gstm7 Gstm2 Txnrd1 Gstk1 Apoa4
Proteolysis	0.18145087	18	1101	1.553697266	Npepps Capns1 Psmd2 Psmd11 Psmb6 Psmb5 Capn1 Psmd13 Ubxn4 Pdia3 Eif3f Cops8 Uggt1 Hist1h2ba Psma5 Dnpep Ctsd Ube2k
Glutathione metabolic process	0.029490401	4	53	7.172413793	Gstm7 Gstm1 Gstm2 Gstk1
Cellular response to oxidative stress	0.013552114	9	230	3.71874063	Nme2 Capn1 Atp2a2 Rps3 Apoa4 Pdk2 Snca Pdcd10 Hnrnpd
KEGG					
Proteasome	2.95E-06	7	47	15.59554108	Psmd2 Psmd11 Psmb6 Psmb5 Psme1 Psmd13 Psma5
Wnt signaling pathway	0.003713261	7	167	4.389164255	Rac1 Rac3 Ppp3cb Camk2g Ppp3cc Ppp3ca Rac2
Cellular senescence	0.151733662	4	174	2.407193532	Ppp3cb Ppp3cc Capn1 Ppp3ca
Valine, leucine and isoleucine degradation	0.170753942	2	56	3.739747095	Hibadh Hibch
Oxidative phosphorylation	8.34E-07	11	133	8.660466957	Atp5e Ndufs4 Ndufb9 Ndufb8 Cox8b Ndufb3 Ndufc2 Cox7a2 Ndufa3 Ndufa7 Ndufb6
CGMP-PKG signaling pathway	0.004122548	7	171	4.286493746	Ppp3cb Ppp3cc Adcy4 Rock1 Ppp3ca Atp2a2 Agtr1b
Gastroc Pathway Analysis					
Pathway_GO Biologicals					
Branched-chain amino acid catabolic process	0.003832993	3	12	38.80319149	Hibadh Acat1 Hibch
Regulation of intrinsic apoptotic signaling pathway in response to DNA damage	0.024606067	3	36	12.93439716	Hnrnpk Mtch2 Rps3
Cellular detoxification	0.01456807	5	108	7.185776202	Cd36 Gstm7 Adh5 Gsr Gstm2
Glutathione metabolic process	0.068842511	3	70	6.651975684	Gstm7 Gsr Gstm2
Response to oxidative stress	0.030079841	9	442	3.160440936	Cd36 Nme2 Camk2g Ndufb4 Adipoq Snca Alad Rps3 Gsr
Proteolysis	0.105232184	19	1799	1.639267678	Ctsd Psmb1 Psmb6 Elane Psme1 Hrg C3 Snca Pdia3 Mtch2 Nsfl1c Alad A2m Rps3 Psma4 Gpi1 Serpinb6a Serpinh1 Serpina1d
KEGG					
CGMP-PKG signaling pathway	1.74E-06	10	171	9.076769939	Ppp1cc Atp1a2 Ppp1cb Slc25a5 Ppp3cb Ppp3cc Mylk Ppp3ca Ppp1ca Calml3
Wnt signaling pathway	9.20E-05	8	167	7.435342082	Rac1 Rac3 Ppp3cb Camk2g Ppp3cc Ppp3ca Rac2 Camk2d
Cellular senescence	0.000119648	8	174	7.136219124	Ppp1cc Ppp1cb Slc25a5 Ppp3cb Ppp3cc Ppp3ca Ppp1ca Calml3

Proteasome	0.0010 68729	4	47	13.209 5971	Psmb1 Psmb6 Psme1 Psma4
Valine, leucine and isoleucine degradation	0.0018 51769	4	56	11.086 62614	Acads Hibadh Acat1 Hibch
Oxidative phosphorylation	0.0001 65741	7	133	8.1690 92945	Ndufa2 Atp5e Ndufa5 Ndufb8 Sdhc Cox5b Cox7a1
