# Supplementary material for: LaminA/C-dependent cellular senescence signaling promotes skeletal muscle atrophy and abnormalities in Parkinson’s disease
Source: Cell Death Dis. 2026 Jun 6;17(1):709. doi: 10.1038/s41419-026-08962-0 (PMC13463094; doi:10.1038/s41419-026-08962-0)
Supplement: Supplementary file 3 [file 41419_2026_8962_MOESM3_ESM.pdf]

Fig2 Q and V

Gas

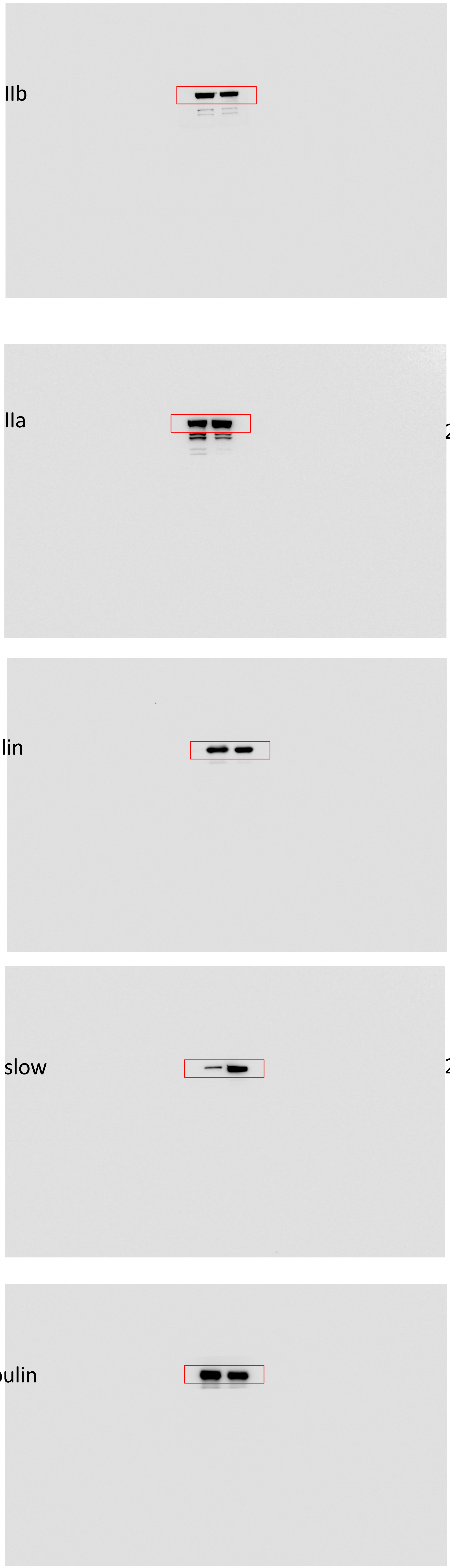

Fig2 Q and V

TA

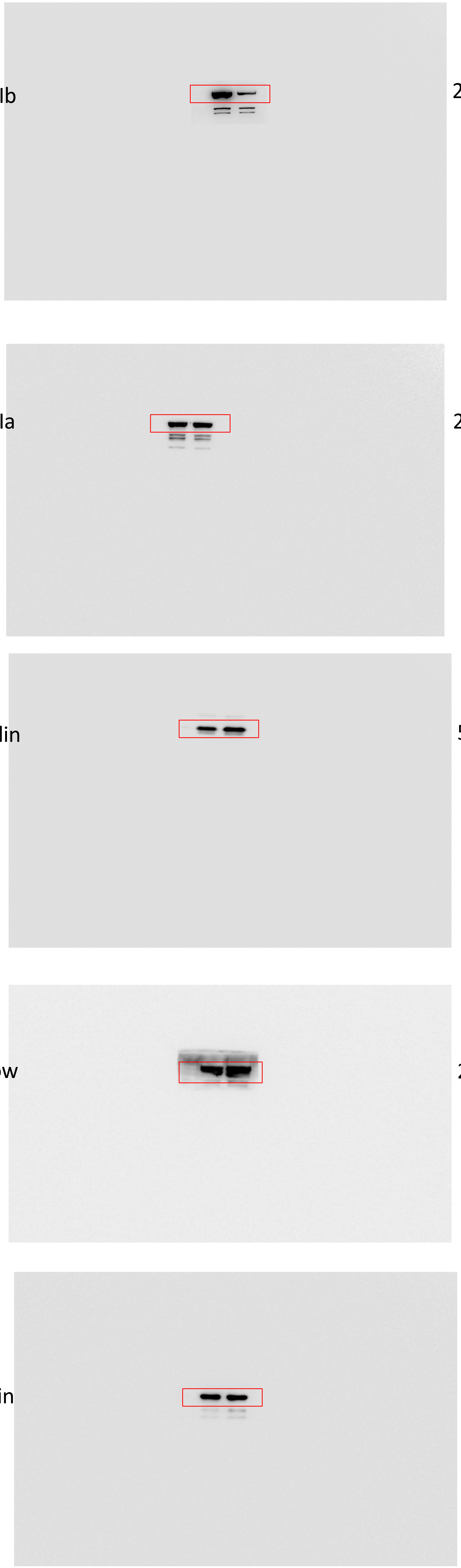

Fig3 G

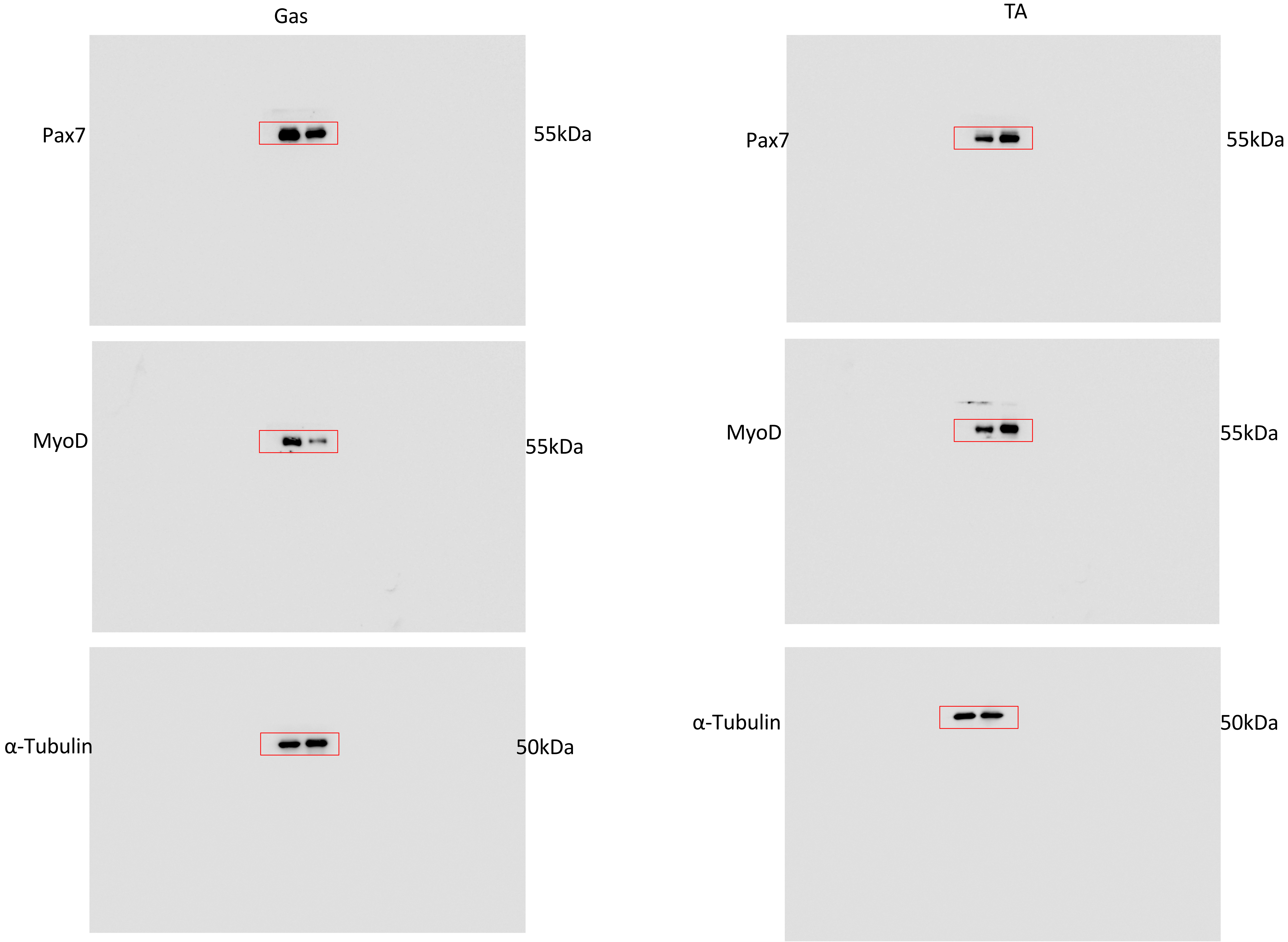
Gas
TA
Pax7
55kDa
Pax7
55kDa
MyoD
55kDa
MyoD
55kDa
α-Tubulin
50kDa
α-Tubulin
50kDa

Fig5 E

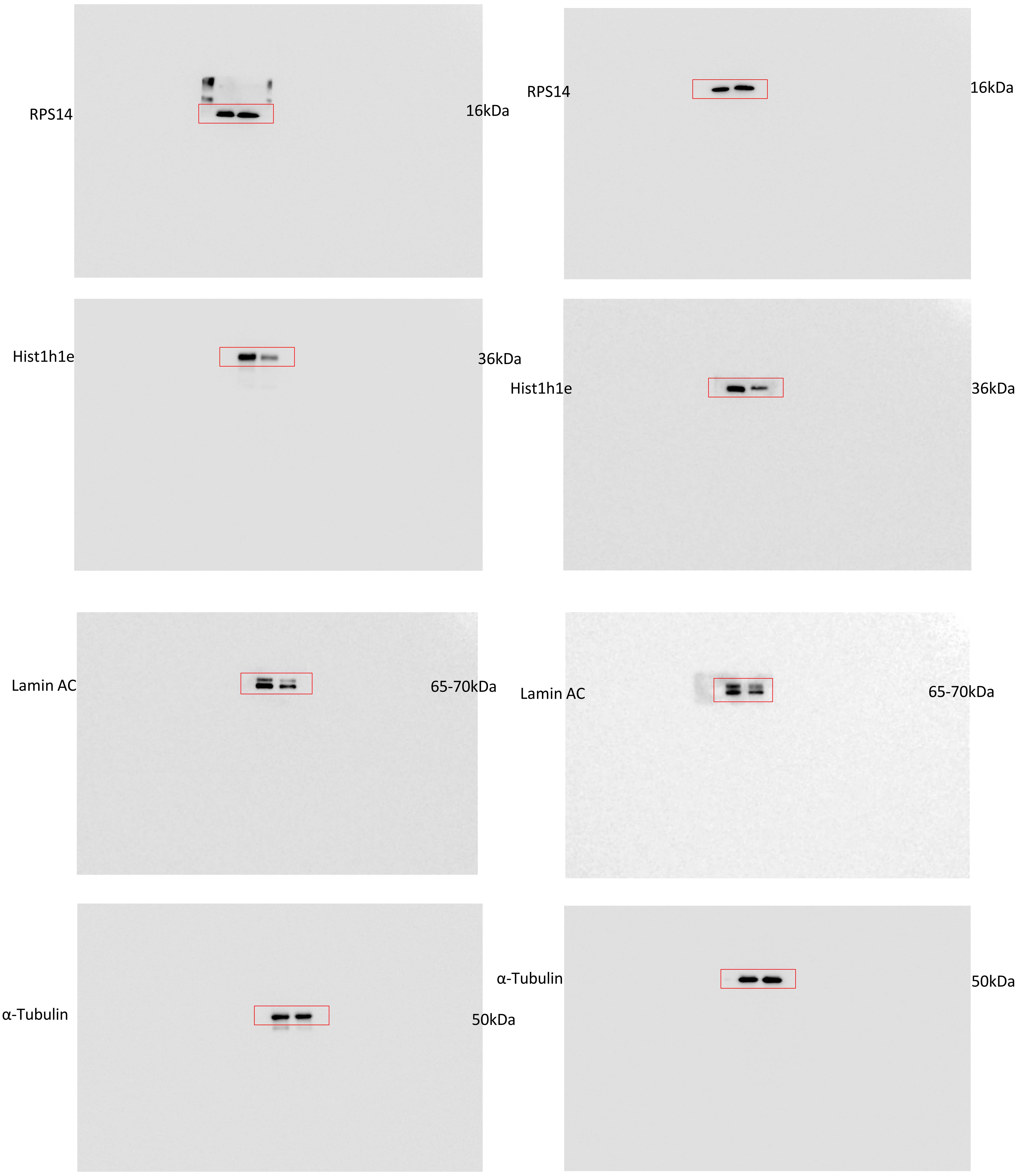
RPS14
16kDa
RPS14
16kDa
Hist1h1e
36kDa
Hist1h1e
36kDa
Lamin AC
65-70kDa
Lamin AC
65-70kDa
α-Tubulin
50kDa
α-Tubulin
50kDa

Fig5 H

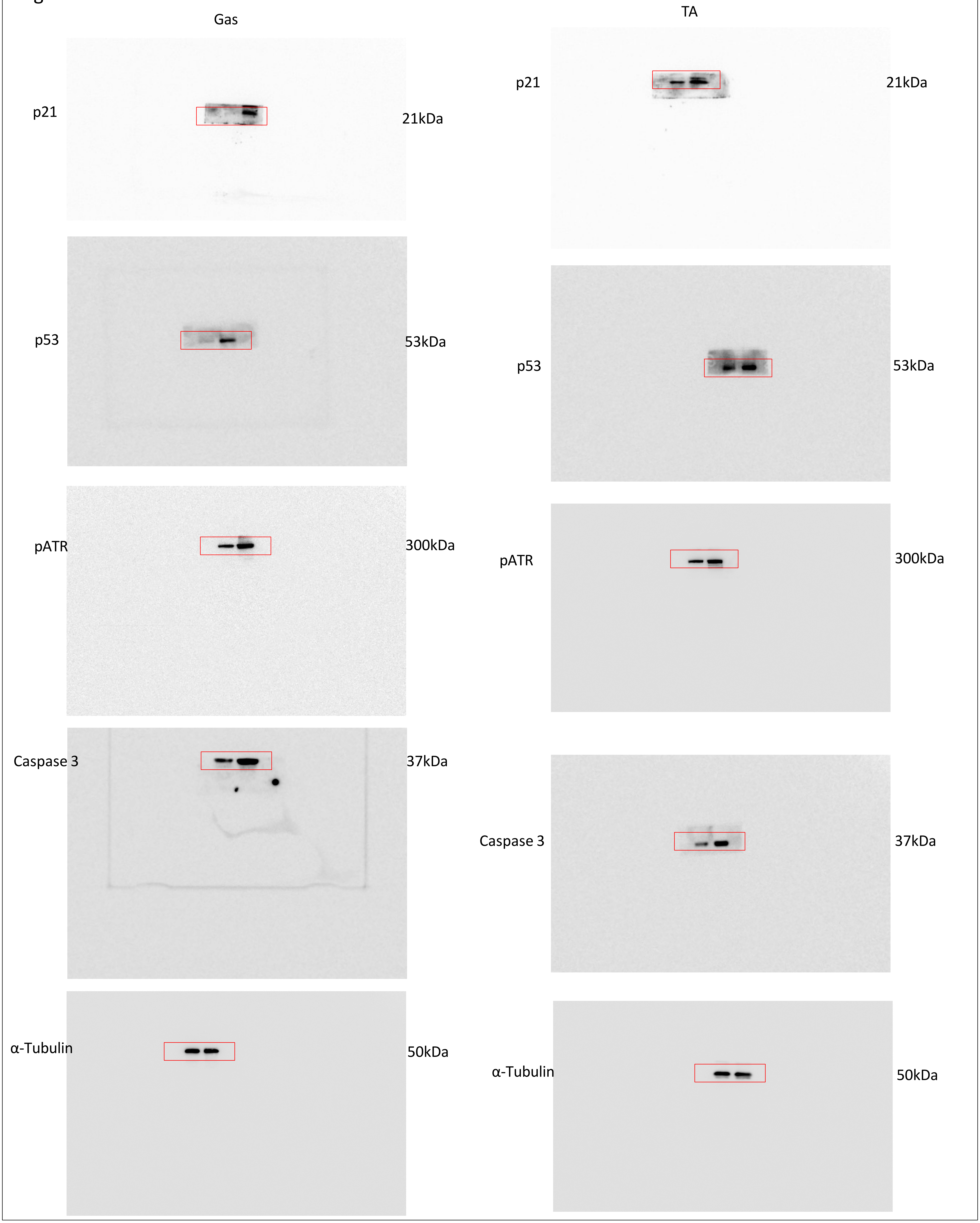
Gas
TA
p21
21kDa
p21
21kDa
p53
53kDa
p53
53kDa
pATR
300kDa
pATR
300kDa
Caspase 3
37kDa
Caspase 3
37kDa
α-Tubulin
50kDa
α-Tubulin
50kDa

Fig7 A

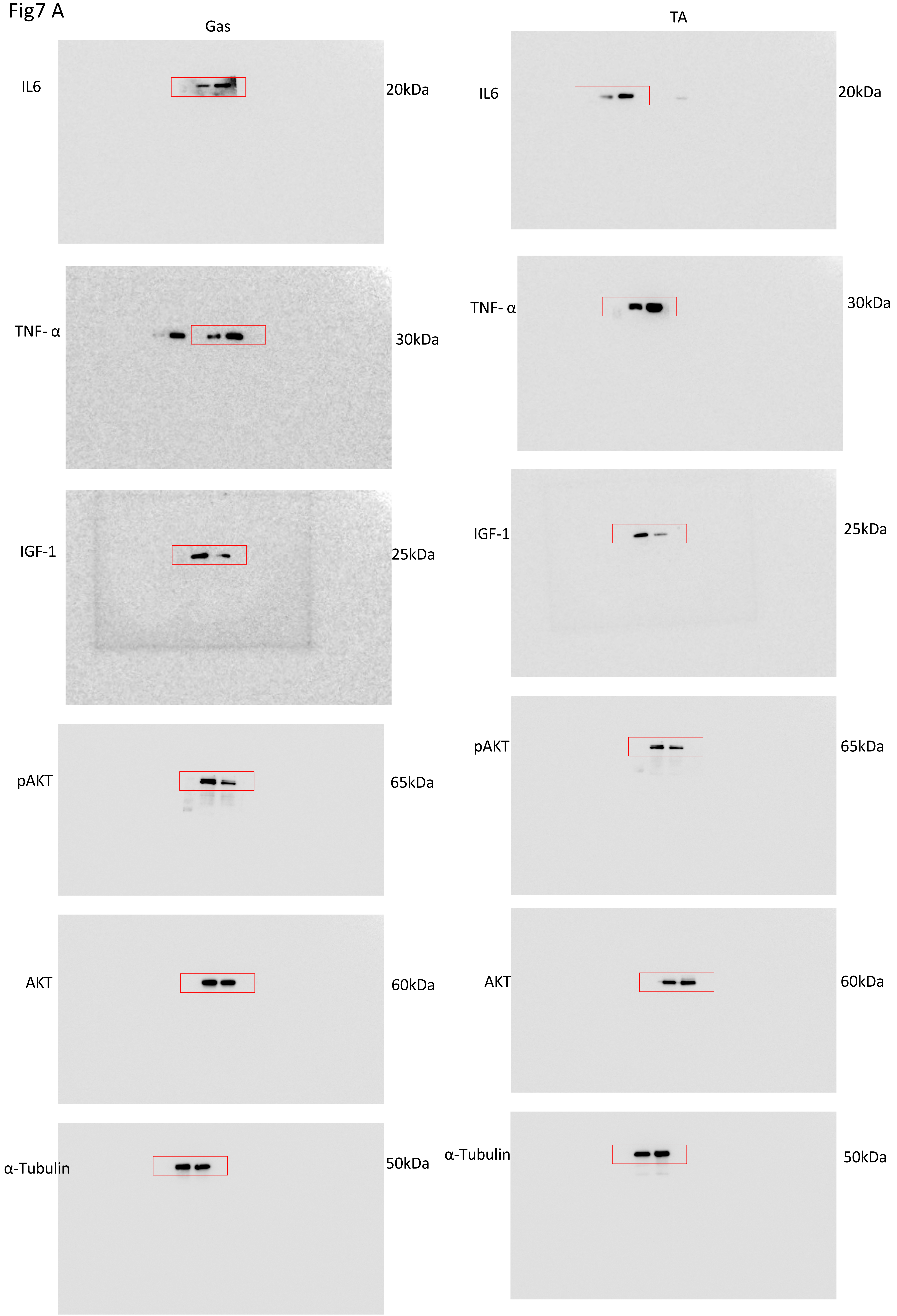
Gas
TA
IL6
20kDa
IL6
20kDa
TNF- α
30kDa
TNF- α
30kDa
IGF-1
25kDa
IGF-1
25kDa
pAKT
65kDa
pAKT
65kDa
AKT
60kDa
AKT
60kDa
α-Tubulin
50kDa
α-Tubulin
50kDa

Fig7 D

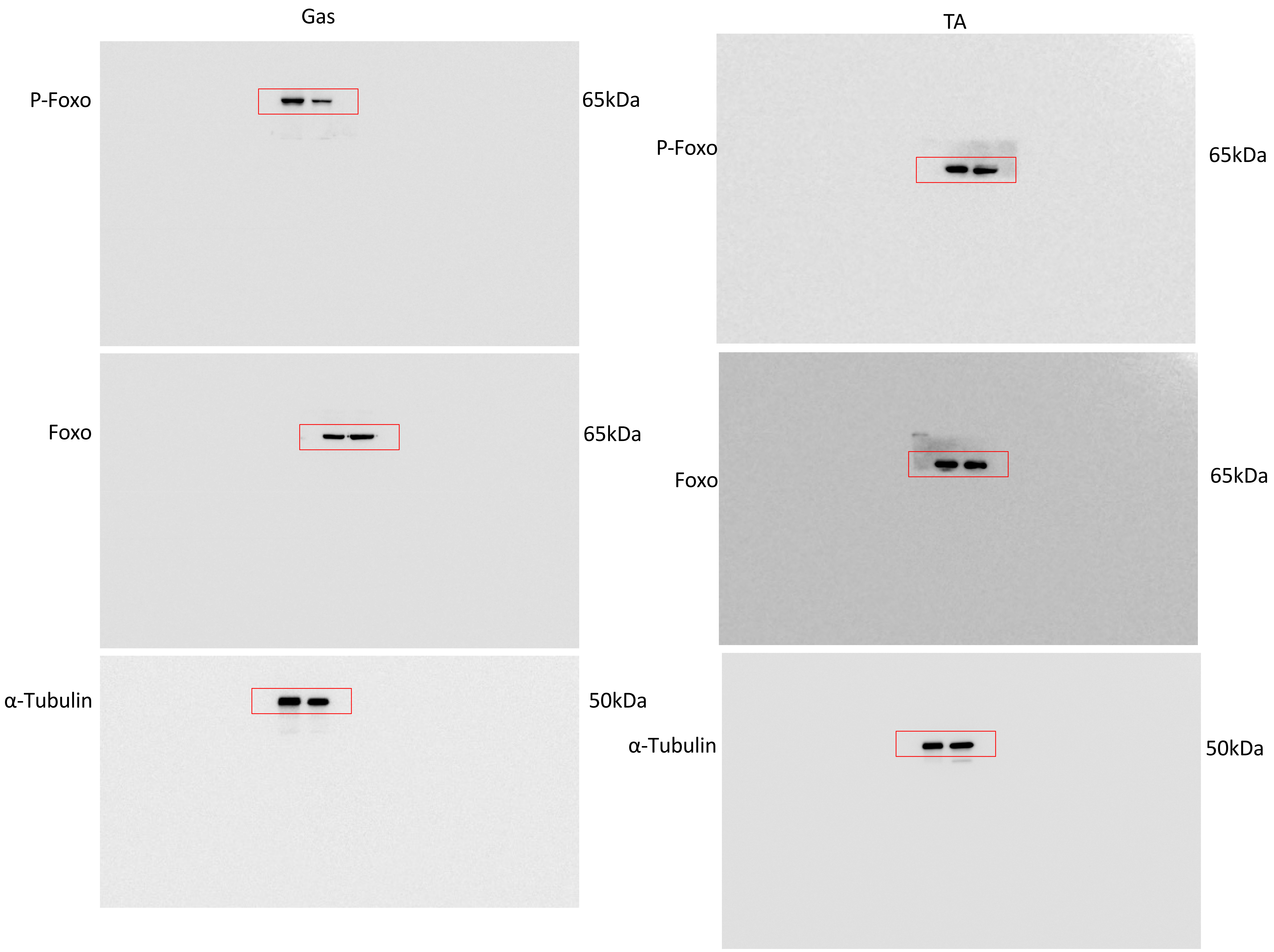
Gas
TA
P-Foxo
65kDa
P-Foxo
65kDa
Foxo
65kDa
Foxo
65kDa
α-Tubulin
50kDa
α-Tubulin
50kDa

Fig7 I and J

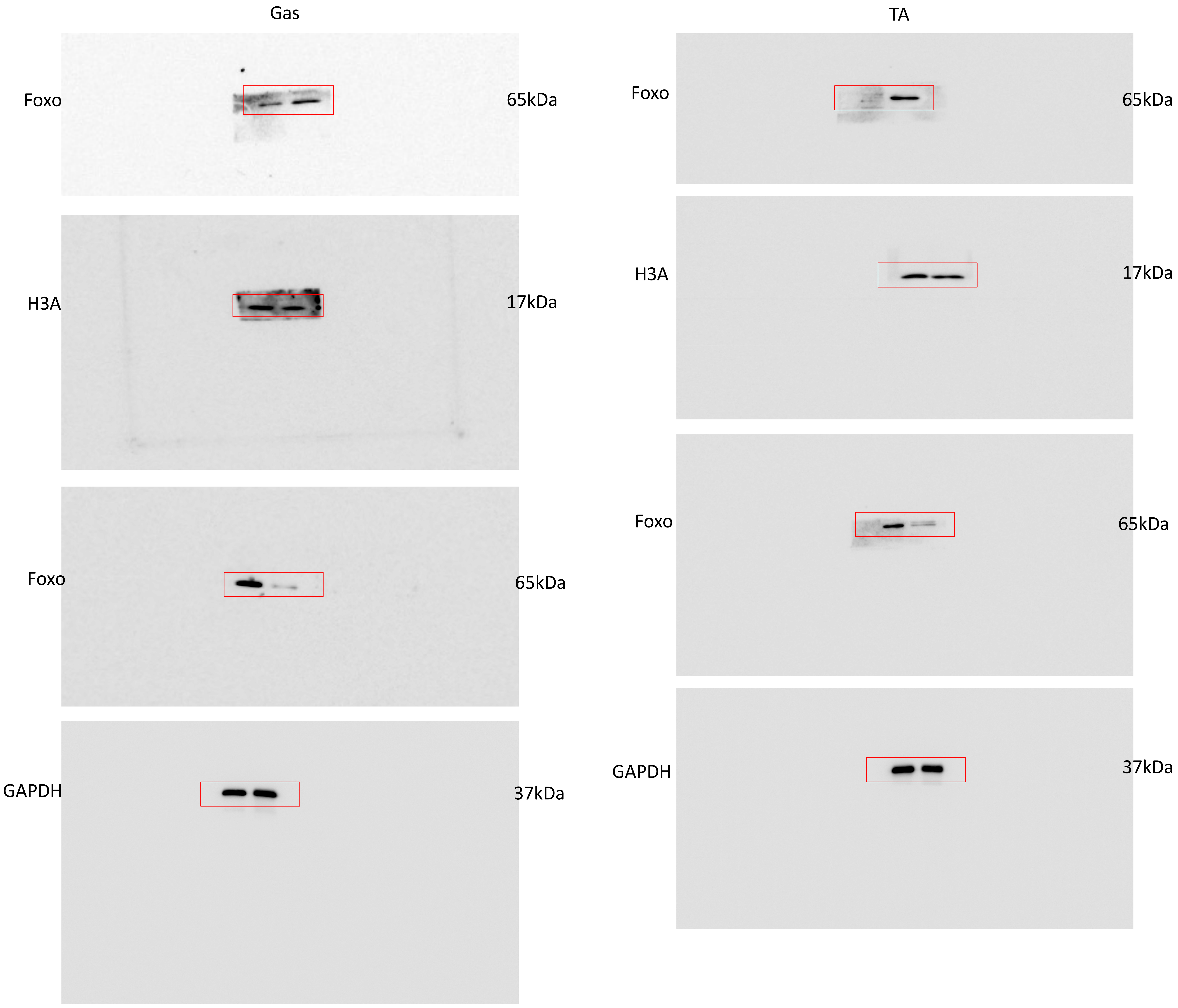
Gas
TA
Foxo
65kDa
Foxo
65kDa
H3A
17kDa
H3A
17kDa
Foxo
65kDa
Foxo
65kDa
GAPDH
37kDa
GAPDH
37kDa

Fig7 M

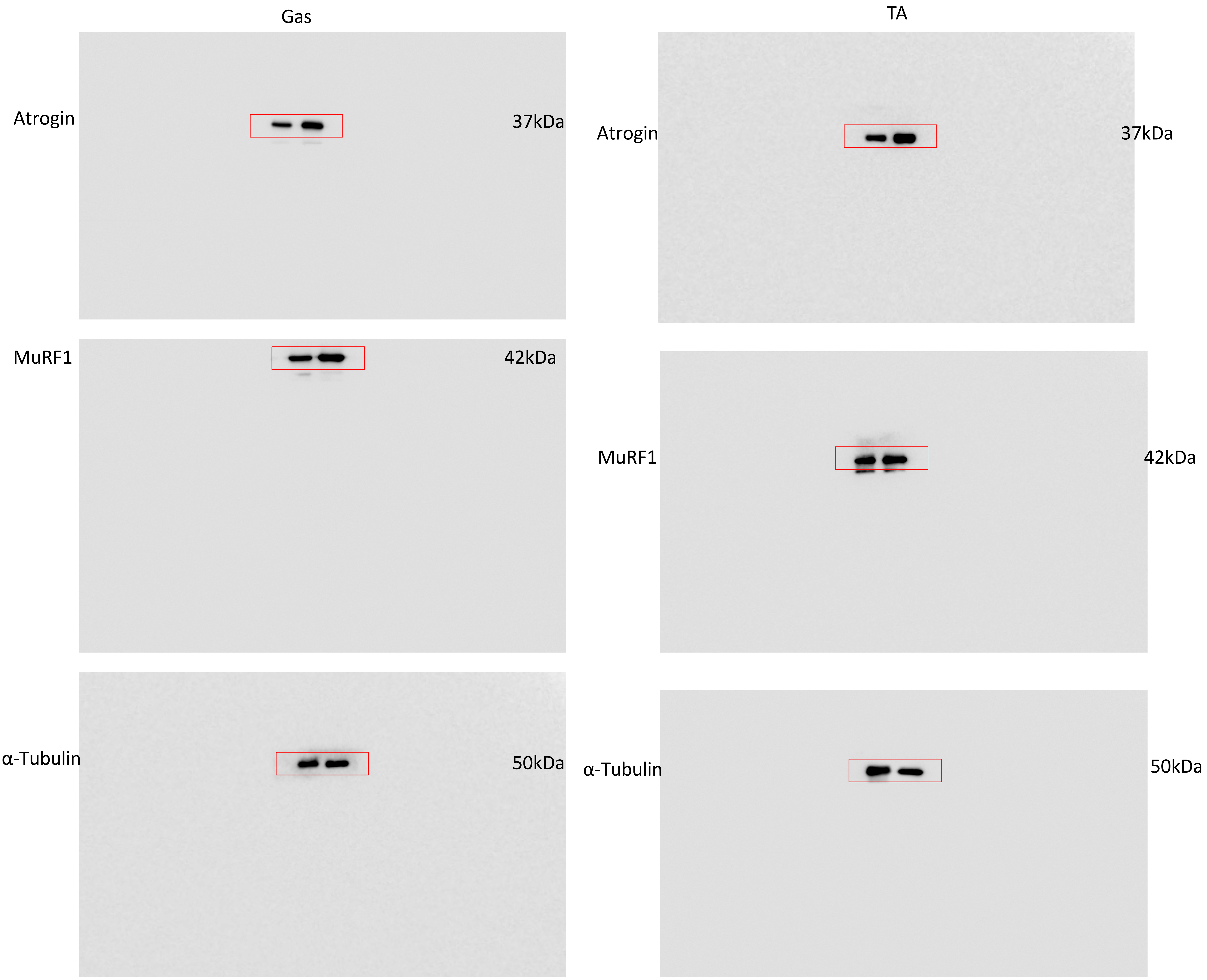

Gas
Atrogin
37kDa
MuRF1
42kDa
α-Tubulin
50kDa
TA
Atrogin
37kDa
MuRF1
42kDa
α-Tubulin
50kDa

Fig7 P Q

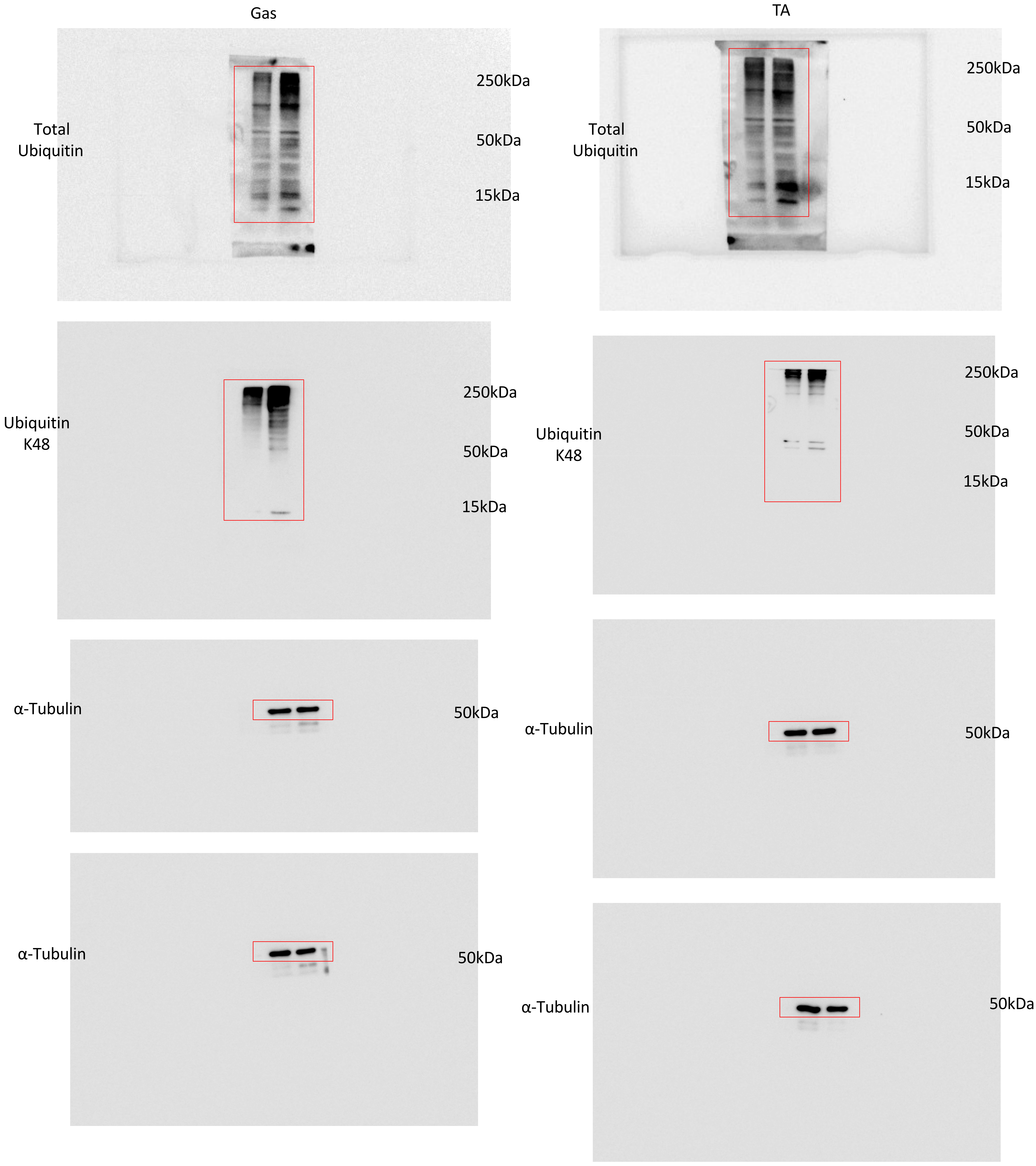

Gas
TA
Total Ubiquitin
250kDa
50kDa
15kDa
Total Ubiquitin
250kDa
50kDa
15kDa
Ubiquitin K48
250kDa
50kDa
15kDa
Ubiquitin K48
250kDa
50kDa
15kDa
α-Tubulin
50kDa
α-Tubulin
50kDa
α-Tubulin
50kDa
α-Tubulin
50kDa

FigS1 V W

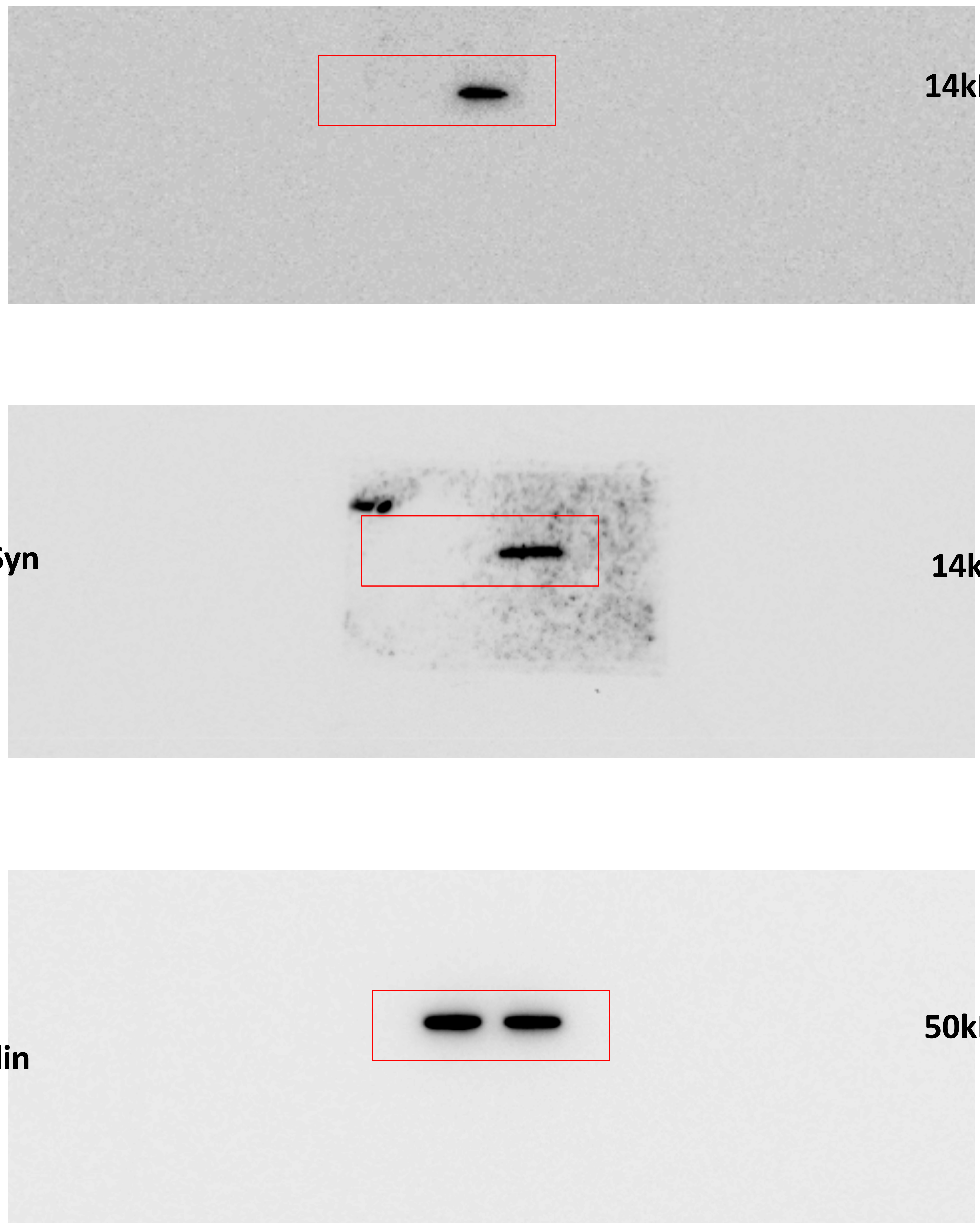

α-Syn 14kDa

P-α-Syn 14kDa

α-Tubulin 50kDa

FigS2 J-M

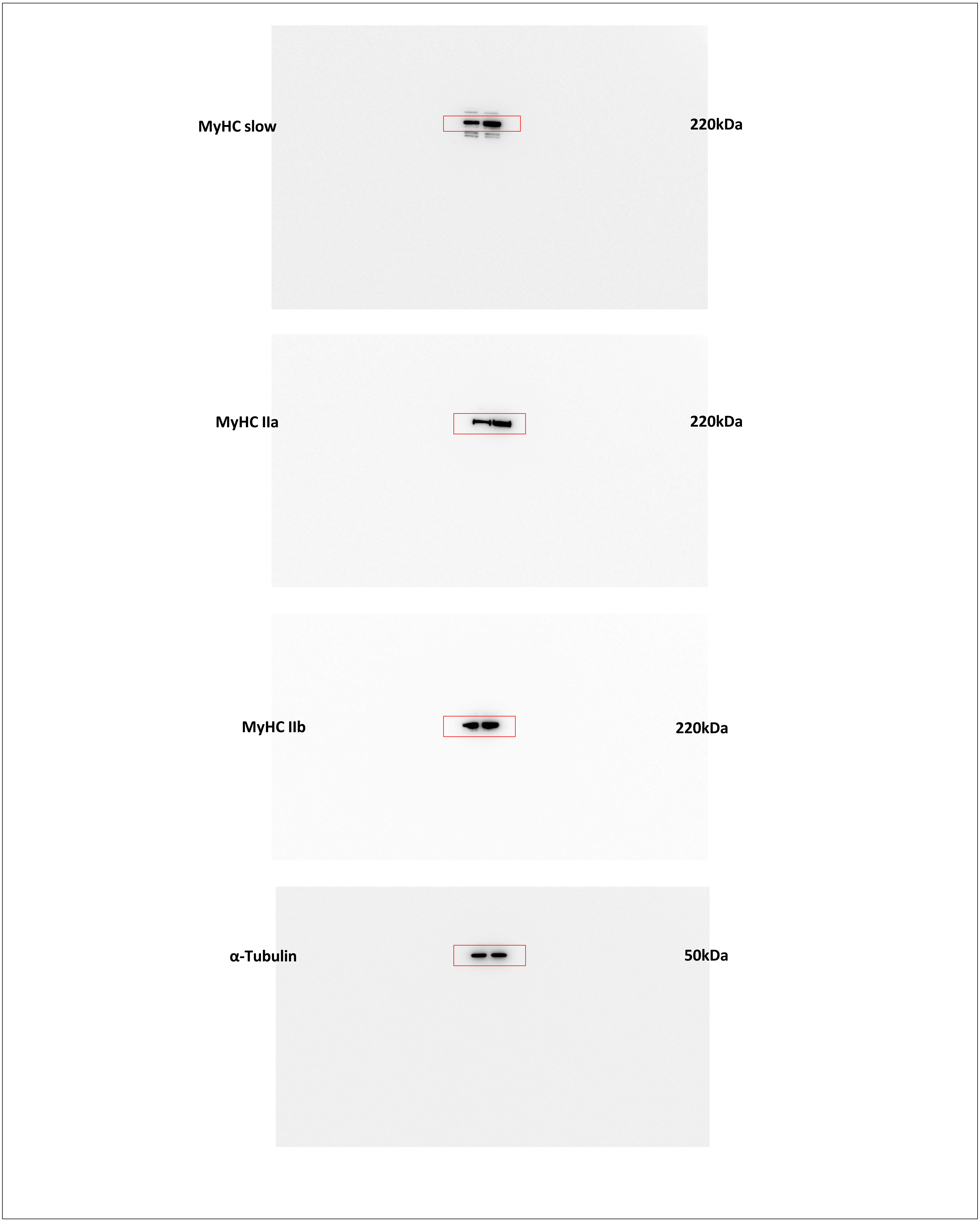
MyHC slow
220kDa
MyHC IIa
220kDa
MyHC IIb
220kDa
α-Tubulin
50kDa

FigS4 E-I

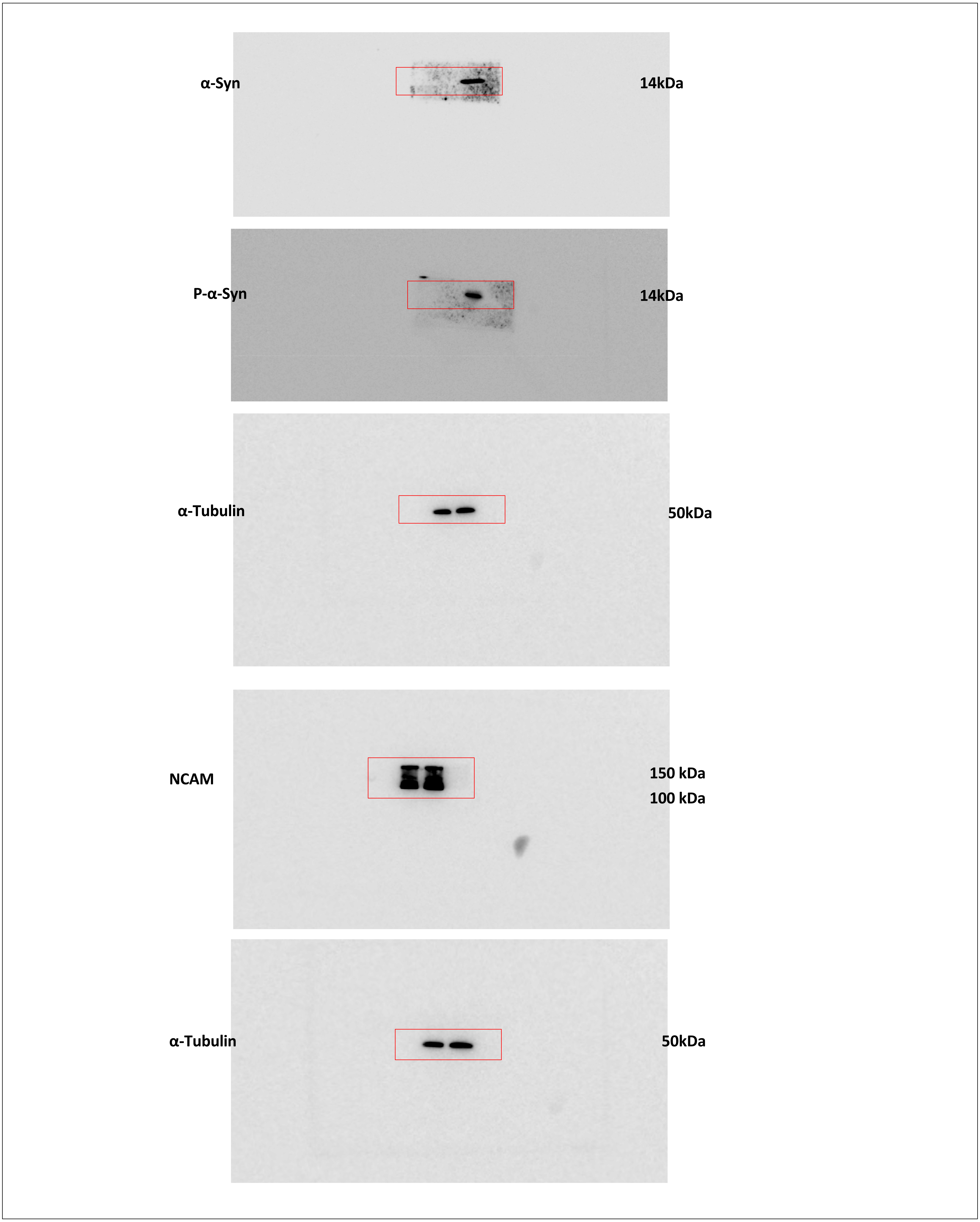
α-Syn
14kDa
P-α-Syn
14kDa
α-Tubulin
50kDa
NCAM
150 kDa
100 kDa
α-Tubulin
50kDa

FigS6 A-D

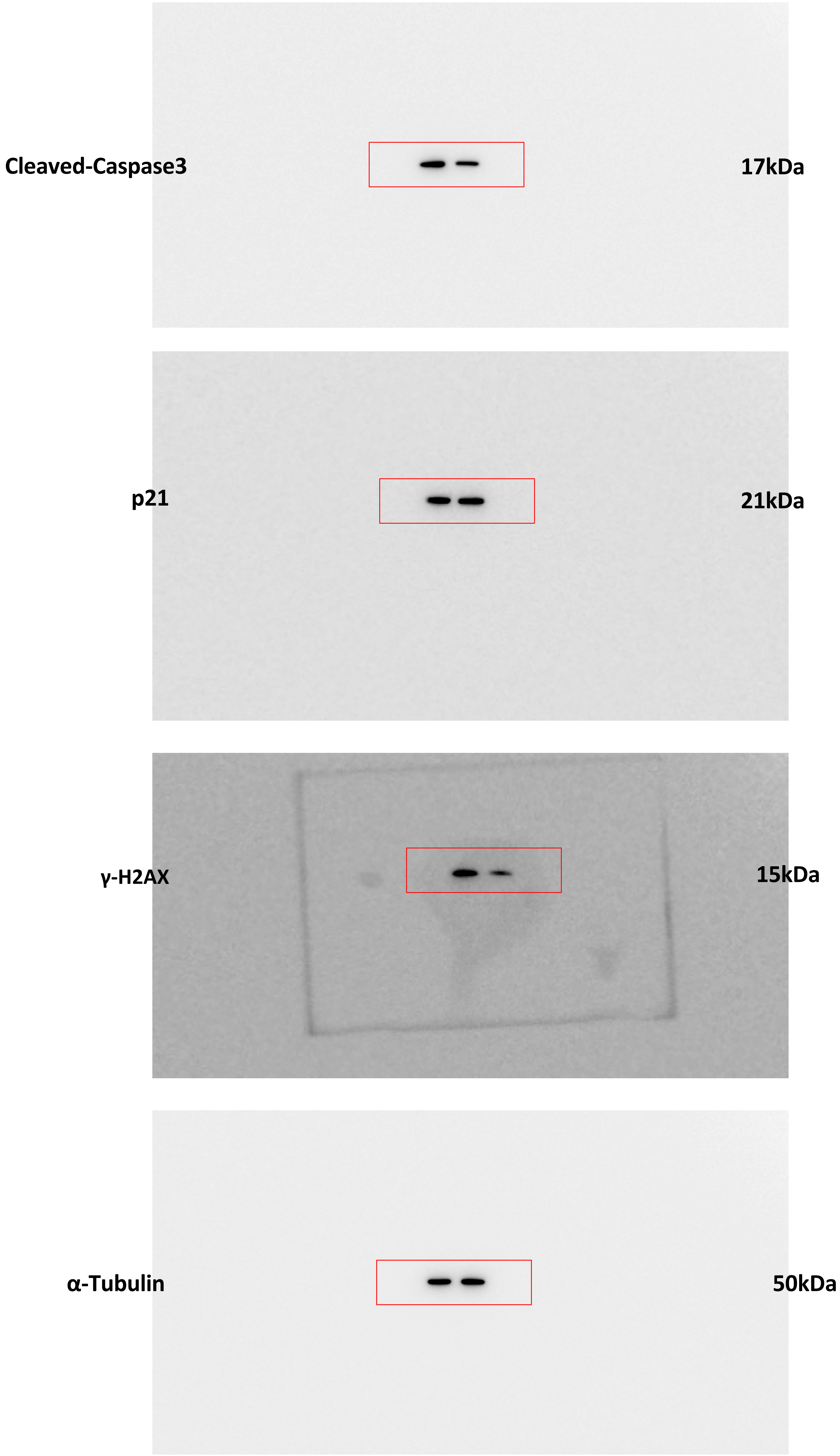
Cleaved-Caspase3
17kDa
p21
21kDa
γ-H2AX
15kDa
α-Tubulin
50kDa
